# Developmentally regulated GTPases: structure, function and roles in disease

**DOI:** 10.1007/s00018-021-03961-0

**Published:** 2021-10-19

**Authors:** Christian A. E. Westrip, Qinqin Zhuang, Charlotte Hall, Charlotte D. Eaton, Mathew L. Coleman

**Affiliations:** 1 Tumour Oxygenase Group, Institute of Cancer and Genomic Sciences, University of Birmingham, Edgbaston, Birmingham, B15 2TT UK; 2 Present Address: Institute of Inflammation and Ageing, University of Birmingham, Edgbaston, Birmingham, B15 2TT UK; 3 Present Address: Neurological Surgery, School of Medicine, University of California, 1450 Third St, San Francisco, CA 94158 USA

**Keywords:** Translation, Ribosome, GTPase, Rbg1, Rbg2, Tma46, Gir2

## Abstract

GTPases are a large superfamily of evolutionarily conserved proteins involved in a variety of fundamental cellular processes. The developmentally regulated GTP-binding protein (DRG) subfamily of GTPases consists of two highly conserved paralogs, *DRG1* and *DRG2*, both of which have been implicated in the regulation of cell proliferation, translation and microtubules. Furthermore, DRG1 and 2 proteins both have a conserved binding partner, DRG family regulatory protein 1 and 2 (DFRP1 and DFRP2), respectively, that prevents them from being degraded. Similar to DRGs, the DFRP proteins have also been studied in the context of cell growth control and translation. Despite these proteins having been implicated in several fundamental cellular processes they remain relatively poorly characterized, however. In this review, we provide an overview of the structural biology and biochemistry of DRG GTPases and discuss current understanding of DRGs and DFRPs in normal physiology, as well as their emerging roles in diseases such as cancer.

**Supplementary Information:**

The online version contains supplementary material available at 10.1007/s00018-021-03961-0.

## Introduction

**Fig. 1:**
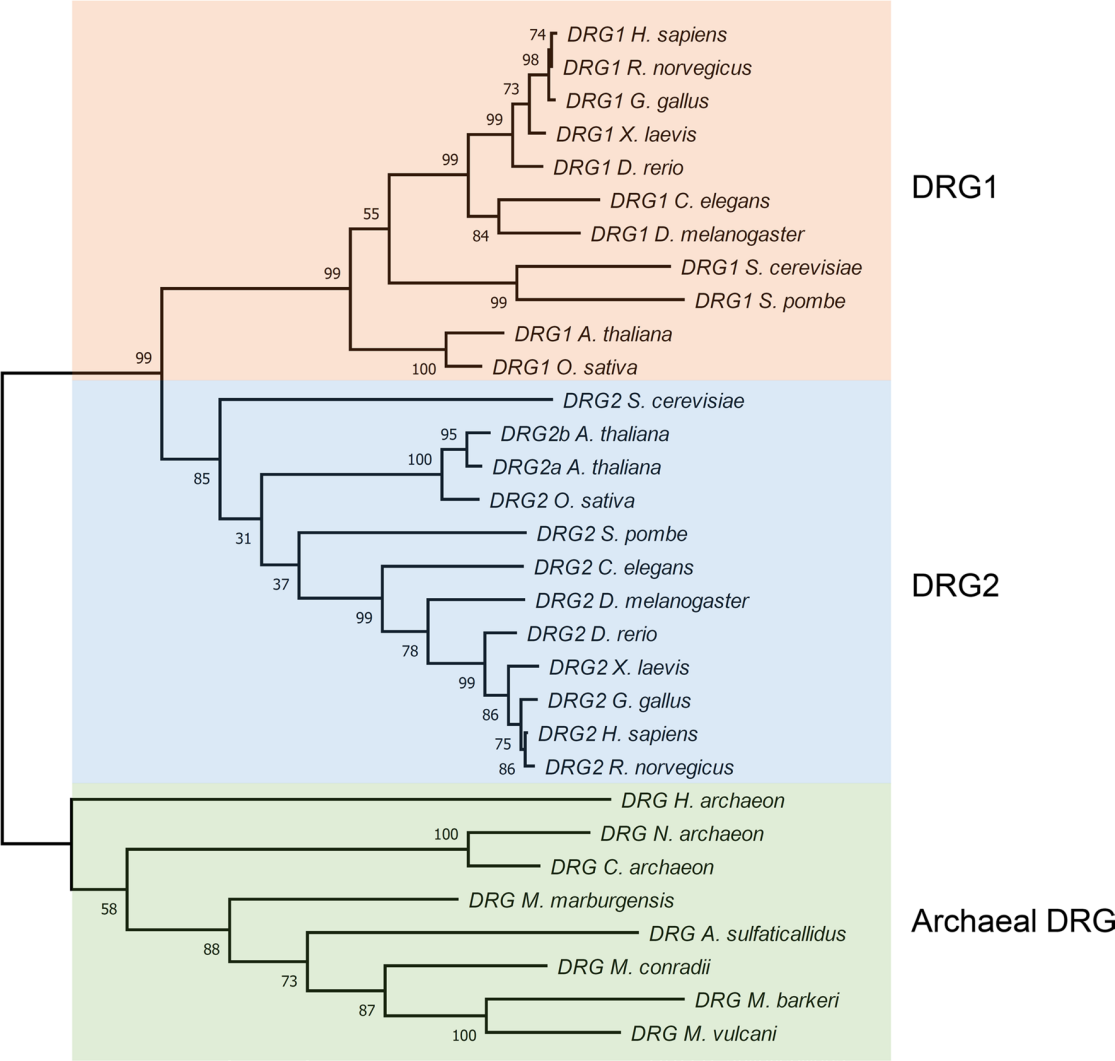
Phylogenetic tree of DRG proteins showing their evolutionary relationships. Sequences were downloaded from NCBI and aligned using the MUSCLE algorithm (a copy of the alignment used can be found in the supplementary file). The phylogenetic tree was estimated using the Maximum Likelihood method, with 500 bootstrap replicates. Bootstrap values are indicated. All analysis was performed in MEGA7. *H.* *sapiens*: *Homo* *sapiens*, *R.* *norvegicus*: *Rattus* *norvegicus*, *G.* *gallus*: *Gallus* *gallus*, *X.* *leavis*: *Xenopus* *leavis*, *D.* *rerio*: *Danio* *rerio*, *D.* *melanogaster*: *Drosophila* *melanogaster*, *C.* *elegans*: *Caenorhabditis* *elegans*, *O.* *sativa*: *Oryza* *sativa*, *A.* *thaliana*: *Arabidopsis* *thaliana*, *S.* *pombe*: *Schizosaccharomyces* *pombe*, *S.* *cerevisiae*: *Saccharomyces* *cerevisiae*, *H.* *archaeon*: *Heimdallarchaeota* *archaeon*, *N.* *archaeon*: *Nanoarchaeota* *archaeon*, *C.* *archaeon*: *Crenarchaeota* *archaeon*, *M.* *marburgensis*: *Methanothermobacter* *marburgensis*, *A.* *sulfaticallidus*: *Archaeoglobus* *sulfaticallidus*, *M.* *conradii*: *Methanocella* *conradii*, *M.* *barkerii*: *Methanosarcina* *barkerii*, *M.* *vulcani*: *Methanolobus* *vulcani*

The developmentally regulated GTP-binding (DRG) proteins are a family of highly conserved GTPases that consists of two closely related paralogs: *DRG1* and *DRG2* (Fig. 1) [1]. *DRG*s appear to be conserved across all eukaryotes and even have homologues in Archaea (Fig. 1) (though not in bacteria) [1]. Whilst there are typically two *DRG* genes in eukaryotes, Archaeal species may have only one copy, which appears to be fairly well distributed throughout the group (Fig. 1). Whether DRG1 or DRG2 is the ancestral protein in eukaryotes is unclear as neither homolog appears to be more or less closely related to the Archaeal DRG protein. DRG1 and DRG2 proteins are highly similar (57% identity in humans), about 40 kDa in size and have a conserved binding partner called DRG family regulatory protein 1 and 2 (DFRP1 and DFRP2), respectively [2]. Though DRG1 and DRG2 are highly similar, their respective DFRPs share only limited similarity (12% identity in humans). DFRP1 and DFRP2 bind to their respective DRG and prevent it from being degraded, likely via the ubiquitin proteasome system [2]. Thus, DRGs and DFRP proteins exist predominantly in complex with each other [3].

**Fig. 2:**
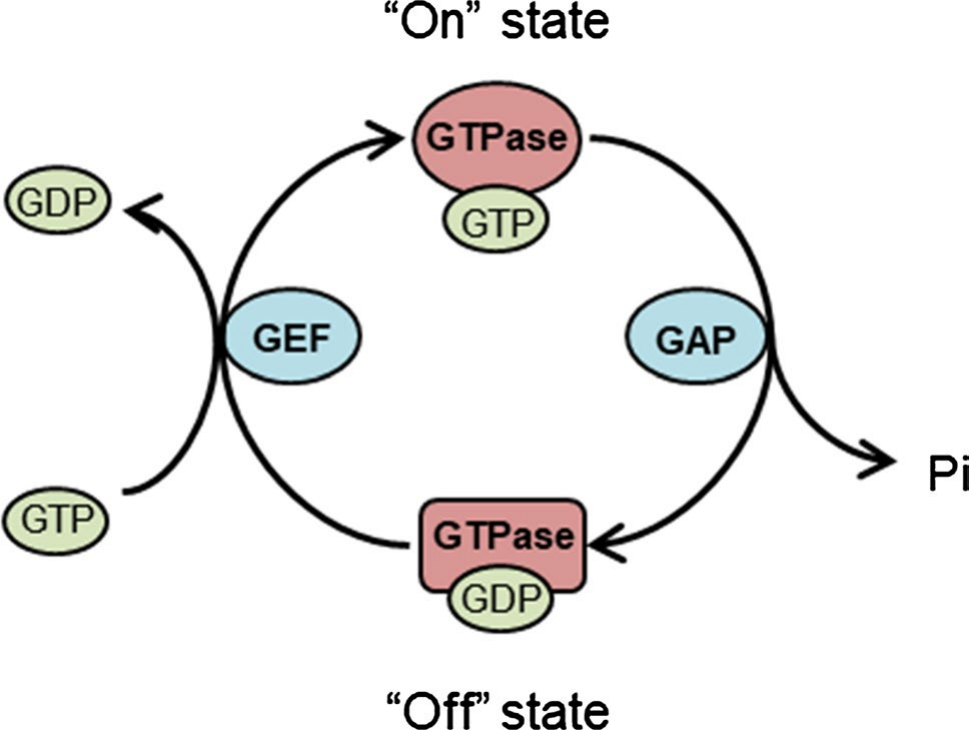
The canonical GTPase cycle. GTPases cycle between an active GTP bound “on” state and an inactive GDP bound “off” state with the help of GAPs and GEFs. GAP: GTPase Activating Protein. GEF: guanine nucleotide exchange factor, Pi: phosphate

Being GTPases, DRG1 and DRG2 belong to the same superfamily of proteins that includes the well-known Ras family of GTPases and the G-protein coupled receptors (large heterotrimeric GTPases) [4]. GTPases form a large superfamily of regulatory proteins that are capable of binding GTP and hydrolysing it into GDP [5]. Members of this superfamily have been implicated in a variety of cellular processes and represent some of the most highly conserved proteins in all of life [4, 6, 7]. The classical mechanism of action for a GTPase is known as the GTPase cycle [5, 8, 9] (Fig. 2). When a GTPase is bound to a molecule of GTP it causes a conformational change in the protein’s structure. In this “on” state, the protein is considered to be active such that it can now interact with its downstream effectors. For the protein to be switched “off”, the bound GTP must be hydrolysed into GDP which can then in turn be exchanged for a new GTP molecule. Thus, GTPases are often described as molecular switches because of their ability to cycle between a GTP bound “on” state and a GDP bound “off” state. This process of GTP binding and hydrolysis is typically slow in most GTPases but is sped up by the action of guanine nucleotide exchange factors (GEFs) and GTPase activating proteins (GAPs) (Fig. 2) [10]. For more detailed reviews of GTPases and their regulation see [5, 9, 10].

The most well-known GTPases are the members of the Ras superfamily. These small GTPases typically function as molecular switches in signal transduction pathways regulating a variety of cellular processes [7]. They are also commonly implicated in diseases such as cancer and as such have been the focus of research for many years [6, 11]. Despite the very large amount of work focusing on GTPases, there are many subfamilies that have remained relatively unexplored. The DRG GTPases represent one such subfamily. Continued research in this field is required to establish clear roles for these proteins. In this review we discuss the structure and function of DRG GTPases plus their binding partners, DFRPs, in normal physiology and in disease.

## History of DRGs

The first *DRG* gene to be identified was *DRG1*. Kumar et al. identified a set of mRNAs that are highly expressed during mouse embryonic brain development but are downregulated in the adult [12]. Sazuka et al. discovered that one of these genes had the necessary residues required for GTP binding, and showed that it could indeed bind to GTP [13]. Thus, they named it developmentally regulated GTP-binding protein (DRG). Subsequently, homologues of this DRG protein were identified in several other organisms [13–15]. However, it was not initially realized that there were in fact two paralogous *DRG* genes. Only in 2000 was it shown that most eukaryotes contain two different copies (some plants have three copies as a result of a recent duplication of the *DRG2* gene: see Fig. 1), now referred to as *DRG1* and *DRG2* [1]. *DRG2* was first identified as a gene whose mRNA transcript is downregulated in SV-40 transformed fibroblasts (though at the time it was not called DRG2) [16]. The significant difference in sequence between the DRGs and other GTPases led to the creation of a new subfamily of GTPases [4].

## Nomenclature

Due to overlapping nomenclature, the DRG GTPases are often confused in the literature with other proteins that also share the acronym ‘DRG’. These include AAA-ATPase diazaborine resistance 1 (DRG1) and differentiation-related gene 1/N-myc downstream regulated 1 (NDRG1). DRG can also refer to dorsal root ganglion. Therefore, it is important to note that these are distinct from the DRG GTPases and are not part of this review.

Furthermore, it should be noted that DRGs and DFRPs also have different names based on the various ways in which they have been identified, as shown in Table 1. For the purpose of this review we shall use the mammalian homologue names unless otherwise stated (Table 1).

**Table 1:** Different names used to refer to DRGs and DFRPs

Mammals	Yeast	Other names
DRG1	Rbg1	NEDD3 (gene name)
DRG2	Rbg2	–
DFRP1	TMA46	Lerepo4, ZC3H15
DFRP2	Gir2	RWDD1

*NEDD3* NPC (neural precursor cell) expressed developmentally downregulated 3, *TMA46* translation machinery associated 46, *Rbg1/2* ribosome binding GTPase 1/2, *RWDD1* RWD domain containing 1, *ZC3H15* zinc finger CCCH-type containing 15, *Lerepo4* likely orthologue of mouse immediate early response erythropoietin 4

## Overview of the OBG/HflX superfamily of GTPases

**Fig. 3:**
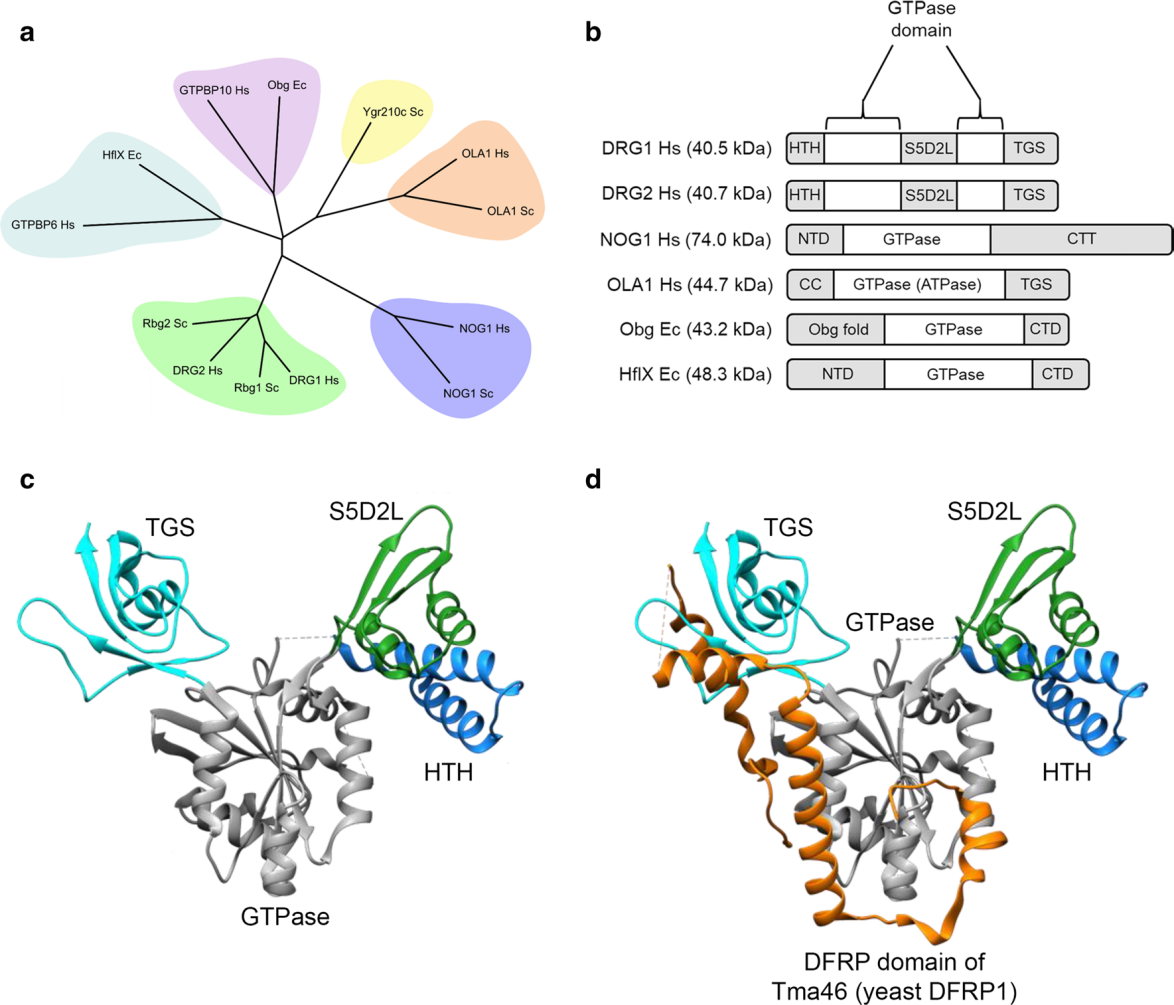
Domain organization of DRG GTPases, conserved members of the OBG-HflX superfamily. **a** Phylogenetic tree of example proteins from the OBG, NOG1, DRG, OLA1, Ygr210 and HflX families. Protein sequences were downloaded from NCBI and aligned using the MUSCLE algorithm. The phylogenetic tree was estimated using the minimal evolution method in MEGA7. **b** Domain organization of some of the GTPases shown in part (**a**). *HTH* helix turn helix, *S5D2L* ribosomal protein S5 domain 2-like domain, *TGS* ThrRS, GTPase, and SpoT domain, *CTD* C-terminal domain, *NTD* N-terminal domain, *CTT* C-terminal tail, *CC* coiled-coil. In both (a) and (b) *Hs*: *Homo* *sapiens*, *Sc*: *Saccharomyces* *cerevisiae*, *Ec*: *Escherichia* *coli*. **c** Crystal structure of Rbg1 on its own and **d** in complex with the DFRP domain of Tma46. The DFRP domain is the region of DFRPs that interacts with DRG proteins. Structure from Francis et al., PDB:4A9A [25]. Images created in Chimera

DRG1 and DRG2 belong to the OBG family of GTPases, named after the founding GTPase Obg (spoOB-associated GTP-binding protein). The OBG family can be split into five subfamilies; Obg, YyaF/YchF/OLA1, Ygr210, Nog1 and DRG (Fig. 3a + b) [4]. The HflX (high frequency of lysogenization protein X) GTPases are another family of GTP binding proteins that are related to the OBG family (Fig. 3a) [4]. Members of the OBG and HflX families are all highly conserved, with some homologues found in all domains of life. Many of the members of the OBG family have roles in ribosome regulation/biogenesis or have been implicated in RNA binding [3, 17–22]. Based on this, it has been suggested that this family represents a group of ancient translation factors [4]. For instance, the bacterial Obg protein has been suggested to function as a ribosome splitting factor that promotes the dissociation of the 70S ribosome into the 30S and 50S subunits [20]. Similar to the OBG family GTPases, HflX proteins have also been implicated in the regulation of translation [21]. In bacteria, HflX has been shown to interact with ribosomes and promote the splitting of ribosomes similar to the function reported for Obg [21]. For the most part, research into the OBG and HflX GTPases has focussed on the bacterial and yeast homologues and it is not yet clear if the homologues from higher eukaryotes have retained an important role in ribosome biogenesis and regulation. For many of these GTPases including DRGs, detailed molecular and cellular characterization is still lacking.

## Structure, biochemistry and regulation of DRGs

### Structure of DRG1/2

Many of the Ras superfamily GTPases are typically small (20–25 kDa), consisting of just a GTPase domain. However, the GTPases in the OBG family are often larger due to the presence of additional domains (Fig. 3b) [23, 24]. For instance, Obg contains a unique N-terminal domain called the Obg fold (Fig. 3b), which has been suggested to function as a protein–protein interaction domain [23]. The crystal structure of Rbg1 (the yeast homologue of DRG1; see Table 1) shows that it has three additional domains that protrude out from the GTPase domain (Fig. 3b + c) [25]. At the N terminus, Rbg1 has a helix-turn-helix (HTH) domain that sticks outwards suggesting a role in mediating interactions (Fig. 3b + c). At the apex of this HTH domain is a highly conserved lysine that is hydroxylated by the 2-oxoglutarate (2OG) dependent oxygenase JMJD7 in higher eukaryotes [26]. DRG2 is also modified at this position [26] (DRG hydroxylation is described in more detail below).

Interestingly, Rbg1 has a unique domain that protrudes out from the GTPase domain and makes contact with the HTH (Fig. 3b + c). The inserted domain is named S5D2L, after “Ribosomal protein S5 domain 2-like” because of its structural homology to this region [25]. This fold is typically found in RNA/DNA binding proteins [27, 28]. A third domain is found at the C terminus, named TGS after the protein families it is found in: ThrRS, GTPase, and SpoT [29]. Whilst the HTH and S5D2L domains appear to be unique to DRG GTPases the TGS is not. Several other members of the OBG family also include a C-terminal TGS domain (Fig. 3b) [30]. In Rbg1 the TGS domain is involved in the interaction with Tma46 (yeast DFRP1) (Fig. 3d, discussed below) and has been suggested to have a role in binding double stranded DNA, in the GTPase YchF [24], though this is unconfirmed. Furthermore, a recent paper has revealed how the Rbg1/Tma46 complex interacts with the ribosome [31]. The HTH and GTPase domains interact with the 60S subunit whilst the S5D2L domain contacts the A-site tRNA [31]. Although a structure has not yet been reported for DRG2, the high sequence conservation between DRGs suggests it is likely to have a similar overall fold.

### DFRP proteins

**Fig. 4:**
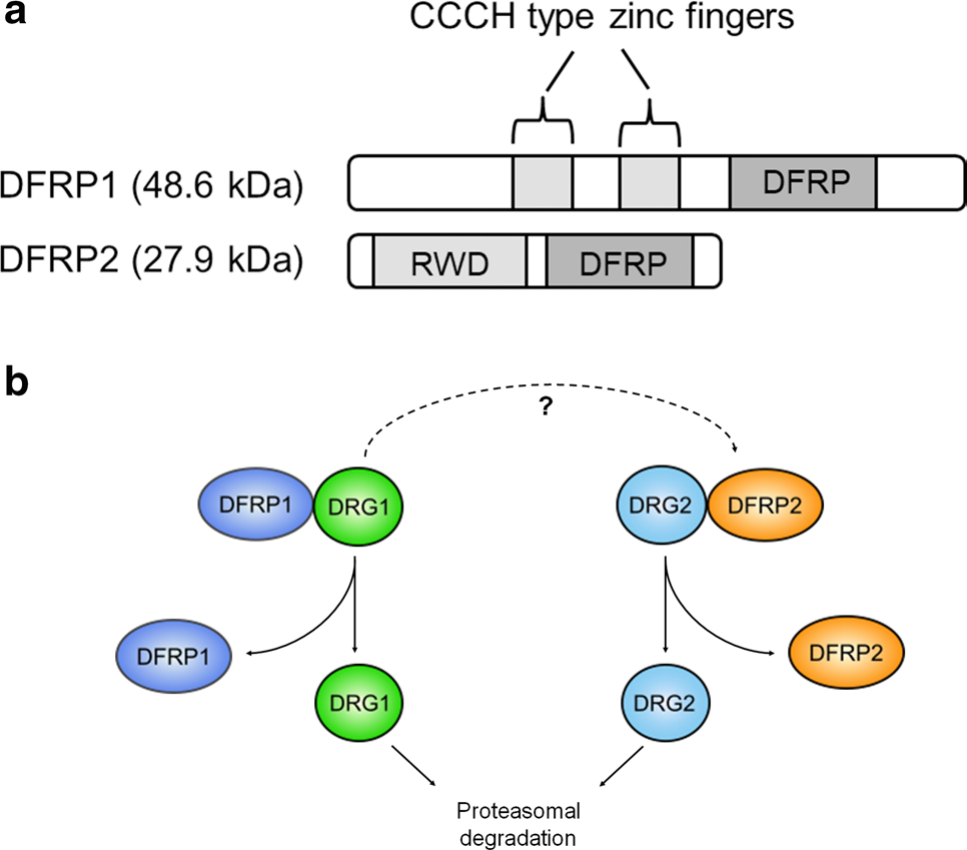
DFRP1/2 domain architecture and regulation of DRGs. **a** Domain architecture of human DFRP1 and 2. The only region conserved between the two proteins is the DFRP domain, the region required for interaction with DRGs. **b** DFRP proteins bind to their respective DRG and prevent it from being degraded, likely via the proteasome. DRG1 has also shown weak binding to DFRP2 when overexpressed (indicated by dashed arrow)

As mentioned in the “Introduction”, both DRG1 and DRG2 have a conserved binding partner called DFRP1 and DFRP2, respectively (Fig. 4a + b) [2]. One function of DFRP proteins is to stabilize the expression of their corresponding DRG. RNA interference-mediated knockdown of either DFRP1 or DFRP2 causes a corresponding decrease in the protein levels of DRG1 or 2, respectively [2, 32]. It has been suggested that this loss of DRG is mediated by proteasomal degradation whereby the binding of DFRP blocks ubiquitination (Fig. 4b) [2]. However, there has been no identification of the ubiquitin site/sites or the E3 ubiquitin ligase responsible. However, it has been proposed that DRG2 is ubiquitinated by a Skp1-Cullin1 containing ubiquitin ligase [33]. Whether this ubiquitination is blocked by DFRP2 is unknown.

Despite the high sequence conservation between DRG1 and DRG2, their binding partners, DFRP1 and 2, respectively, are strikingly different: DFRP1 is 48 kDa in size, whilst DFRP2 is only 28 kDa (Fig. 4a). The only conserved region between the two proteins is the region responsible for interacting with their cognate DRG, termed the DFRP domain (Fig. 4a) [2]. The structure of Rbg1 included the C-terminal fragment of Tma46: It revealed that the DFRP domain wraps around Rbg1 in an extended conformation, making contacts with both the TGS and GTPase domain (Fig. 3d) [25]. It is possible that the DFRP domain only adopts this structure when interacting with DRGs, which may be consistent with an earlier report suggesting that DFRP2 is an intrinsically disordered protein [34].

As previously mentioned, DFRP1 and DFRP2 are considerably different proteins. Besides the DFRP domain, DFRP1 has two conserved CCCH-type zinc fingers that are predicted to interact with RNA (Fig. 4a). Meanwhile, the smaller DFRP2 protein has an N-terminal RWD domain (Fig. 4a). The RWD domain is so named because of the proteins that share this fold: ring-finger containing proteins, WD-repeat-containing proteins and yeast DEAD (DEXD)-like helicases [35]. The RWD structure may also be related to ubiquitin-conjugating enzymes, though it is unlikely that it has any similar enzymatic activity as the catalytic cysteine residue is not usually conserved [35]. Although the function of the RWD domain is not fully understood, it is thought to function in protein interactions [35].

Although many Archaeal species appear to possess a *DRG* gene (Fig. 1) it is not yet clear whether these microorganisms also have DFRP homologs. However, we do note the existence of a protein in *archaeon* (Accession Number A0A482SSL1) that has an assigned C-terminal DFRP-like domain, which raises the possibility that DFRP-like proteins may also be conserved in these species. Further work is required to determine whether these archaeal DFRP candidates bind and regulate the corresponding DRG in a manner similar to that described in higher eukaryotes.

Given the large difference between DFRP1 and DFRP2 it is possible that the emergence of DFRPs has helped DRG1 and DRG2 to functionally diverge from each other. For this hypothesis to be correct, it would be necessary for DRGs to have maintained binding specificity to their respective DFRP. Indeed, Daugeron et al. showed that DRG1 has specific binding for DFRP1 whilst DRG2 has specific binding for DFRP2 [3] (Fig. 4b). However, it has been reported that DRG1 and DFRP2 can interact weakly when overexpressed [36]. Whether this interaction is a consequence of overexpression or is physiologically relevant in the right context, is not clear; however, Ishikawa et al. suggested it may be an artefact of overexpression [37].

The degradation of DRGs when not bound to their respective DFRP proteins suggests that they might exist primarily in complex with each other. This is supported by evidence suggesting that DFRPs are the most abundant interactors of DRGs [3]. As such, it is highly likely that DFRPs are of considerable importance for DRG function. Despite this, the role of DFRPs in DRG biology is often overlooked. Whether or not DFRP proteins exist outside of this complex is unknown. Interestingly, knockdown of DRGs has also been shown to cause some decrease in the levels of DFRPs, consistent with potential co-regulation of expression [32, 37]. Because of the intimate relationship between DRGs and DFRPs, we shall discuss the functions of both here in this review.

### GTPase activity of DRGs

As members of the GTPase superfamily, DRG1 and 2 both contain a typical GTPase domain that is similar to other OBG family GTPases [25]. The structure of this domain is universally conserved across GTPases, and consists of roughly 5 alpha helices and a 6 stranded beta sheet [5] (Fig. 3c). In addition, this domain has 4–5 highly conserved sequence motifs called G motifs that are responsible for coordinating the binding and hydrolysis of GTP [8]. These motifs are highly conserved between *DRG* homologues (See Supplementary material).

To date the majority of work on DRG GTPase activity has been focused on DRG1. The first reported GTPase activity for DRGs came from *Arabidopsis* *thaliana* (at) orthologues. O’Connell et al. showed that both atDRG1 and atDRG2 could bind and hydrolyse GTP, albeit at a slow rate [38]. Subsequently, Francis et al. assayed the activity of Rbg1 and human DRG1, and showed that both had quite a slow rate of GTP hydrolysis [25]. However, Perez-Arellano et al. reported much higher activity for human DRG1 [39]. This may be explained by the greater amounts of potassium ions used in their assays. Indeed, Ash et al. predicted that metal cations (potassium or sodium) could increase the GTPase activity of many GTPases, with the DRG family predicted to be enhanced specifically by potassium ions [40]. Indeed, Perez-Arellano et al. showed that the activity of DRG1 was strongly stimulated by potassium, but not sodium [39]. Hence, earlier reports of DRG GTPase activity did not take into account the requirement for potassium ions in their GTPase assays. Although DRG2 has not been shown to be stimulated by potassium ions, its high sequence similarity to DRG1 suggests it may be regulated in a similar manner. Although the evidence for a positive role of potassium in supporting DRG activity is relatively clear, at least in vitro, its importance in regulating the GTPase activities of related Obg/HflX family members requires clarification. For example, the E.coli ObgE GTPase appears not to require potassium for activation [22].

Many GTPases also require the presence of additional factors to speed up the slow hydrolysis and exchange of bound GTP/GDP. As outlined in the introduction, these include GAPs that stimulate the hydrolysis of GTP, and GEFs, that stimulate the exchange of GDP for GTP (Fig. 2) [10]. However, DRGs have been shown to bind and hydrolyse GTP without the need for GAPs or GEFs [25, 39]. This is consistent with other members of the OBG family of GTPases that also do not have these classical regulatory proteins [30]. This may be because these factors have not been identified yet, or these GTPases may be utilizing other mechanisms to regulate their GTPase activity. Furthermore, OBG family GTPases typically have faster GTP/GDP exchange kinetics, perhaps explaining why they do not require GEFs [30].

Interestingly, the GTPase activity of DRG1 was shown to be stimulated by its binding partner DFRP1 [39]. Although DFRP1 increases the thermal stability of DRG1 and its affinity for potassium ions [39], the mechanism by which this increases GTPase activity is not clear. It is unlikely that DFRP1 functions as a classical GAP as it does not bind to the active site of the GTPase domain and so cannot provide any residues to enhance GTPase activity. Furthermore, DFRP1 binds DRG1 in the absence of GTP, which is at odds with GAP proteins [25]. Whether DFRP2 also stimulates DRG2 GTPase activity is unknown.

The GTPase activity of DRG1 can also be regulated by phosphorylation. DRG1 was identified as a potential substrate of the kinase STK16 (Serine/Threonine Kinase 16) [41]. STK16 phosphorylates DRG1 on a conserved threonine immediately after the G2 motif. Mutating this threonine to a phosphomimetic aspartate causes a reduction in the GTPase activity of DRG1 whilst not affecting GTP binding [39]. Interestingly, this threonine is not conserved in DRG2, suggesting that this phosphorylation event may be relevant to a DRG1 specific function.

The conformational change that GTPases undergo upon GTP binding regulates their interaction with other proteins that are commonly referred to as ‘effectors’ [5]. In OBG and the related HflX GTPases that have additional domains, it has been suggested that GTP binding may cause a large change in structure that causes the domains protruding from the GTPase fold to undergo a conformational rearrangement [5, 42]. The structure reported for Rbg1 showed the protein in a GTP unbound state [25]. As such, it is currently unknown what sort of conformational change DRGs might undergo when bound to GTP. To date the only GTPase-dependent interaction reported for DRGs is the interaction between Rbg1 and ribosomes (discussed below) [3]. Furthermore, no GTP-dependent DRG2 interactors have been reported.

It should also be noted that some members of the OBG family have been reported to bind and hydrolyse ATP [43]. The protein Obg-like ATPase 1 (OLA1), which is the human orthologue of YchF, has ATPase activity due to changes in its G4 motif [43]. Interestingly, it has been suggested that Rbg1 might also be capable of hydrolysing ATP [31], raising the possibility that DRGs could potentially hydrolyse nucleoside triphosphates in addition to GTP.

### Hydroxylation of DRGs

DRGs were recently identified as being targeted by other PTMs in addition to phosphorylation. DRG1 and DRG2 are both hydroxylated by the 2OG-dependent oxygenase, JMJD7 [26]. Protein hydroxylation is the addition of an oxygen atom to an amino acid creating a hydroxyl group [44–46]. 2OG-oxygenases catalyse hydroxylation reactions using the Krebs cycle intermediate 2OG as well as Fe(II) and oxygen as cofactors [44–46]. JMJD7 hydroxylates a lysine residue that is at the apex of the HTH domain in DRG1 and DRG2 (Fig. 3c). This hydroxylation site is highly conserved across all DRG orthologues. In addition, *Drosophila* *melanogaster* JMJD7 is capable of interacting with and hydroxylating human DRGs, suggesting that this modification is highly conserved and important for the function of DRGs [26].

Interestingly, the amount of hydroxylation is not identical in DRG1 and DRG2: Hydroxylation of DRG1 is more abundant and more responsive to JMJD7 expression than DRG2 [26]. Whether these observations are consistent with JMJD7-mediated hydroxylation imparting differential biological specificity on DRG1 and DRG2 is not yet known.

Unlike some other hydroxylase substrates (e.g. eRF1), DRG hydroxylation also appears to be incomplete in cells [26], raising the possibility that JMJD7 activity and DRG hydroxylation might be regulated. Although signals that modulate JMJD7 activity have not yet been identified, the activity of some 2OG oxygenases is sensitive to the microenvironment. For example, the dependence on oxygen levels makes 2OG oxygenases suitably poised to act as sensors of hypoxia, as is the case for hydroxylases that target the transcription factor Hypoxia Inducible Factor 1α (HIF1α) [44, 46, 47]. Whether JMJD7 activity and DRG hydroxylation is also regulated by changing oxygen levels is unknown.

Hydroxylation events are often involved in regulating interactions [48]. The location of the hydroxylation site on the solvent exposed apex of the HTH fold suggests that this modification may also be involved in regulating interactions of DRGs. In keeping with this, Markolovic et al. suggested that hydroxylation of DRG2 could promote an interaction with RNA [26]. Interestingly, the DRG hydroxylation site shares similarities with eRF1 (eukaryotic release factor 1), which is also hydroxylated at the apex of an HTH motif involved in RNA binding [49]. Furthermore, in a recent paper showing the structure of Rbg1 in complex with the ribosome, the HTH domain is making contact with the 60S subunit [31]. Whether the hydroxylation of DRGs is required for this interaction or plays some other function at the ribosome is unknown.

### Cell biology of DRGs and DFRPs

DRG and DFRP proteins have been implicated in several different biological processes, including protein translation, microtubule regulation and cell growth, which we review here in more detail.

### Expression of DRGs and DFRPs

Early characterization of *DRG1* showed that it is highly expressed in the mouse developing brain and subsequently downregulated in the adult [50]. During murine development, *DRG1* is upregulated in tissues such as the brain, spinal cord, liver and thymus [50]. Therefore, based on its expression pattern Sasuka et al. suggested DRG1 may be involved in proliferation and/or differentiation of neuronal cells [50]. Analysis of *DRG1* transcript levels in *Xenopus* *laevis* revealed a similar pattern of expression in the developing central nervous system (CNS) [15]. Similarly, *Xenopus* *DRG2* showed comparable expression with *DRG1* in development; high expression in the developing CNS, such as the eyes and brain [51]. Analysis of *DFRP1* mRNA levels in *Xenopus* development showed an almost identical pattern to that of *DRG1*, which might be consistent with the two proteins co-operating in their function [2]. Currently, it is unknown if *DFRP2* also shares a similar pattern of expression with *DRG2* during development.

DRGs are also expressed in most adult tissues and various cell lines, albeit to a variable extent; for example, human DRG1 (but not DRG2) is very highly expressed in testis (GTEx Portal). Perhaps consistent with an evolutionary conserved role of DRGs in growth and fertility, expression analysis in plants showed DRG1 and 2 have highest levels in the reproductive organs and growing tissues [52, 53].

Overall, the expression analysis described above supports a role for DRGs in regulating cellular growth and proliferation. Consistent with this, both DRG/DFRP complexes have been implicated in the regulation of translation.

### Ribosomes and translation

The DRG GTPases are most closely related to the OBG family [4]. Members of this family of GTPases have roles in ribosome biogenesis and translational regulation. For instance, the NOG1 GTPase localizes to the nucleolus, the site of ribosome biogenesis, and regulates the production of the 60S ribosomal subunit [18]. Meanwhile, the human orthologues of Obg have been implicated in mitoribosome biogenesis [54]. However, deletion of *Rbg1* and 2 in yeast showed no effect on the total number of ribosomes [3]. Furthermore, DRGs are not strongly expressed in the nucleoli and instead show a more diffuse cytoplasmic expression with some partial expression in the nucleus [26, 55, 56]. Hence, it is likely that these proteins are not directly involved in ribosome biogenesis.

**Fig. 5:**
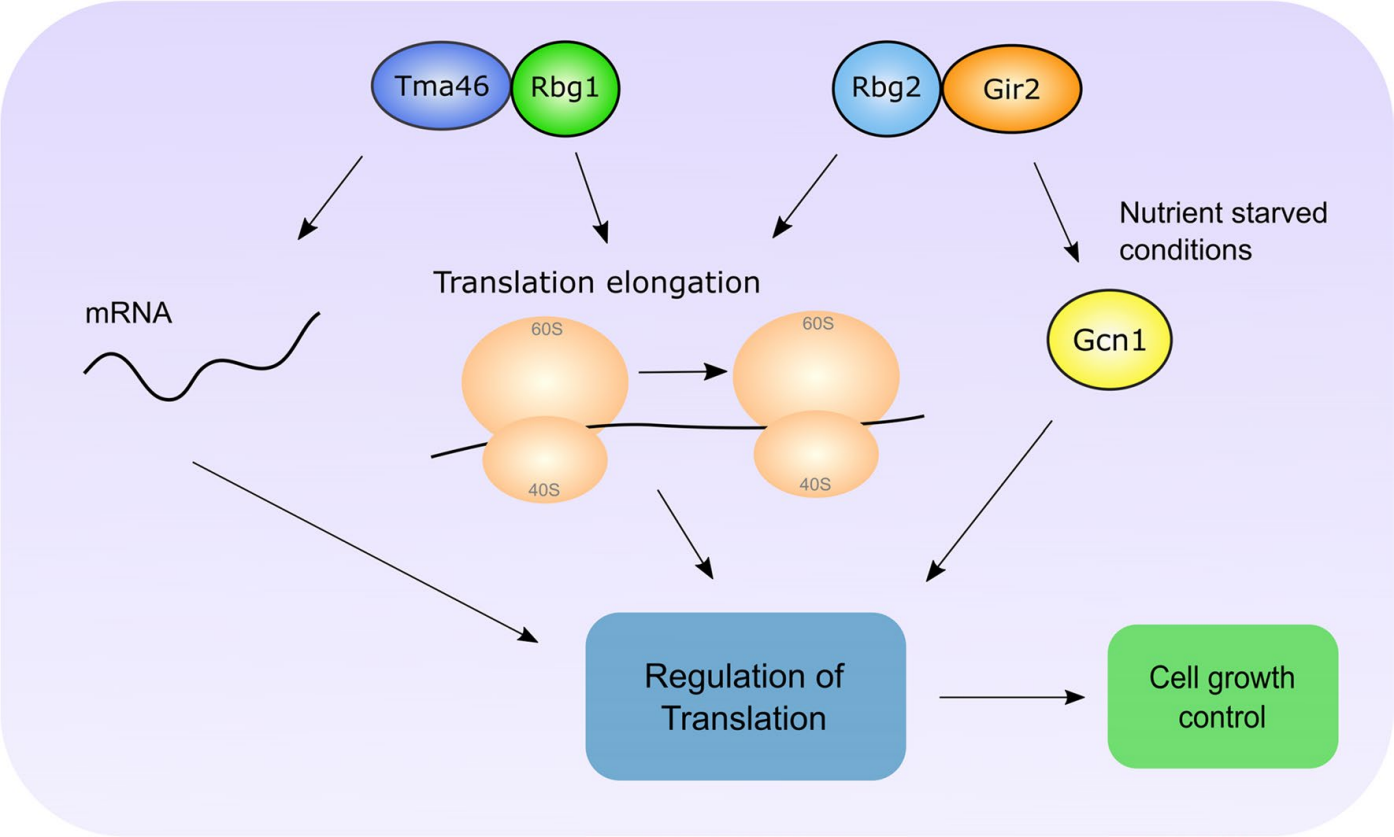
Roles of Rbg1/2 and Tma46/Gir2 in yeast. The Rbg1/Tma46 complex has been implicated in the regulation of translation elongation and mRNA. Rbg2 and Gir2 have been reported to interact with Gcn1, and may regulate translation in response to nutrient starved conditions

DRGs and DFRPs have, however, been implicated in the regulation of translation. DRGs and their binding partners have been shown to associate with ribosomes in yeast (Fig. 5). Wout et al. suggested that Rbg1 could interact with Gir2 (yeast DFRP2) and both could co-sediment with polysomes [36]. Similarly, Daugeron et al. reported that Rbg1 and Tma46 can associate with ribosomes, particularly polysomes, consistent with a role in regulating protein synthesis [3]. This is further supported by the identification of Tma46 in a screen for proteins that are associated with the eukaryotic translation machinery [57].

Deleting the TGS domain of Rbg1 reduces its interaction with ribosomes without affecting the ribosomal association of Tma46 [25]. This suggests that Tma46 binds to the ribosome and recruits Rbg1 through its association with the TGS domain of Rbg1 [25]. Indeed, a recently reported structure of the Rbg1/Tma46 complex bound to a ribosome shows the CCCH-type zinc fingers of Tma46 mediating an interaction with the 40S RNA [31]. This type of zinc finger is also found in mRNA binding proteins [58]. Consistent with the possibility that the Rbg1/Tma46 complex interacts with mRNAs, Tma46 has been identified as a yeast mRNA binding protein in several unbiased screens [59–61].

Although the initial findings suggested that Gir2 can associate with polysomes [36], subsequent work suggests that the Rbg2/Gir2 complex is less ribosome associated compared with the Rbg1/Tma46 complex (Fig. 5) [3, 32]. However, Rbg2 and Gir2 are still likely to interact with ribosomes and have been implicated in regulating translation.

Gir2 can interact with Gcn1 (General Control of Amino-Acid Synthesis 1-Like 1) (Fig. 5), which is an important regulator of translation in response to amino acid starvation [3, 36]. Gcn1 interacts with and activates the eIF-2 kinase Gcn2 (General control non-derepressible 2) under amino acid starved conditions [62]. Interestingly, Gcn2 has an RWD domain that is required for its interaction with Gcn1 [63]. Thus, Gir2 might also bind Gcn1 via its RWD domain. Ishikawa et al. suggested that, in yeast, overexpression of Gir2 reduced cell proliferation under reduced amino acid conditions [37]. This may be caused by Gir2 sequestering Gcn1 away from Gcn2, preventing its activation. Furthermore, the association between Rbg2/Gir2 and Gcn1 is increased during amino acid starvation [37]. As such the Rbg2/Gir2 complex may be involved in regulating translation in response to stress conditions, such as nutrient deprivation (Fig. 5). Whether the interaction between Gir2 and Gcn1 is conserved in higher eukaryotes is unknown. Interestingly, a recent report describes the structure of two collided ribosomes bound by Gcn1 and the Rbg2/Gir2 complex [64]. Though the resolution was not high enough to visualize the full Rbg2/Gir2 complex, Pochopien et al. were able to fit the already available structure of Rbg1 into their model. They suggested that Rbg2 interacts with the region next to the A site, similar to the binding site of other translation GTPases. Furthermore, the RWD domain of Gir2 was suggested to make contact with the C terminus of Gcn1, consistent with the previous reports suggesting that these two proteins interact [3, 36, 37, 64]. Based on these results, Pochopien et al. suggested that Gir2 could be preventing Gcn2 activation by competing for binding to the C terminus of Gcn1. The Rbg2/Gir2 complex may be recruited to the stalled ribosome to promote translation elongation before activation of Gcn2 occurs. This would be consistent with the results of Zeng et al. (discussed below) who suggest Rbg1 and Rbg2 promote efficient translation elongation. It is currently unclear how the Rbg2/Gir2 complex might promote translation elongation. However, because they interact on the ribosome by the A site tRNA, they could be involved in stimulating peptide bond formation or translocation. More detailed biochemical and structural work is needed to address this.

In yeast cells single deletion of either *Rbg1*, *Rbg2*, *Tma46* or *Gir2* does not cause a significant growth defect [3]. Even more surprisingly, combined deletion of either both DRG homologues (*Rbg1* and *Rbg2)* or both DFRP homologues (*Tma46* and *Gir2)* also does not cause a significant growth defect [3], despite both complexes having been implicated in translation. This may be consistent with DRGs and DFRPs only regulating translation of specific mRNAs under normal conditions. An alternative explanation could be that these proteins are involved in regulating bulk translation in response to stress, which would be consistent with the proposed role of Gir2 in amino acid starvation [37]. Of course, these two possibilities are not necessarily mutually exclusive.

Using a genetic screen to identify essential genes in the context of Rbg deficiency, Daugeron et al. identified a conserved RNA helicase, Slh1 [3]. Deletion of *Slh1* in combination with deletion of *Rbg1/2* or *Tma46/Gir2* causes a reduced growth phenotype [3]. In yeast, Slh1 is involved in supressing the translation of non-poly (A) tail mRNAs and is an important component of the ribosome quality control pathway [65, 66]. The human homolog is called Activating Signal Co-integrator Complex 1 Component 3 (ASCC3) and is also involved in the regulation of translation as well as the repair of alkylated and UV damaged DNA [67–69]. Like Rbg1, Slh1 co-sediments with ribosomes and upon deletion of *Rbg1*, *Rbg2* and *Slh1*, ribosome sedimentation profiles showed a decrease in the number of polysomes and an increase in the amount of 80S ribosomes [3]. This suggests that the triple mutant cells have a defect in translation, consistent with the observed reduced growth phenotype of the cells.

More recently a paper has taken the first steps towards elucidating the function of the Rbg1/Tma46 complex at the ribosome [31]. Zeng et al. monitored yeast ribosome dynamics in a genome wide manner using 5P-Seq, a technique that measures the production of 5’ monophosphate mRNA sequences produced by the 5’ exonuclease digestion of mRNAs that are being translated. Using 5P-Seq, Zeng et al. showed that the Rbg1/Tma46 complex is likely involved in promoting translation elongation through motifs that are prone to ribosome stalling, specifically lysine/arginine rich sequences. Slower translation elongation observed by Zeng et al. was most prominent in the strain with triple Rbg1, Rbg2 and Slh1 loss of function compared to the strain with deletion of *Rbg1* only. This suggests there is some functional redundancy between the three factors. Zeng et al. proposed a model whereby the DRG/DFRP complexes promote translation elongation though mRNA sequences that cause ribosome pausing/stalling. When the DRG/DFRP complexes are inactivated the Slh1/ASCC3 factor can rescue the stalled and collided ribosomes through the ribosome quality control pathway. Thus, when both DRG/DFRP complexes and Slh1/ASCC3 are inactivated, the cells are less able to overcome the damaging effect of stalled ribosomes. Prior work used rescue experiments in the triple deletion yeast strains to elucidate the importance of the different domains in Rbg1, Tma46 and Gir2. Whereas re-expression of wildtype Rbg1 was sufficient to restore normal growth, a GTPase inactive Rbg1 mutant was not [3]. This is consistent with a GTPase domain mutant showing reduced ribosome binding [3] and suggests the interaction is important for function. Furthermore, Francis et al. also showed that deleting the HTH or TGS domain of Rbg1 failed to rescue the triple mutant growth phenotype [25]. On the other hand, deletion of the S5D2L domain showed a modest ability to rescue cell growth, suggesting it is not as important for Rbg1 function as the HTH and TGS domains. Reconstitution of a triple knockout strain (Δ*Tma46*, Δ*Gir2*, Δ*Slh1*) with wildtype Tma46 was also sufficient to rescue the growth phenotype, whereas a Tma46 variant with a mutation in the second zinc finger was not [3]. Although both zinc fingers of Tma46 are highly conserved, this would suggest the second zinc finger is more important for its role in promoting growth. Interestingly, deletion of the RWD domain in Gir2 also prevented it from rescuing the growth phenotype [3].

The requirement of the GTPase activity of Rbg1 and the zinc fingers of Tma46 for rescue of the growth phenotype are consistent with their role in the ribosome interaction [31]. Similar to the structure of Rbg2/Gir2 with collided ribosomes, the structure reported by Zeng et al. shows Rbg1 binding to the GTPase association centre, making contact with the ribosome through its GTPase, HTH and TGS domain, whilst the S5D2L domain makes contact with the A site tRNA. Furthermore, the zinc fingers of Tma46 make contact with the 40S subunit, potentially explaining why they are important for ribosome association and rescue of the triple deletion yeast strain [31].

**Fig. 6:**
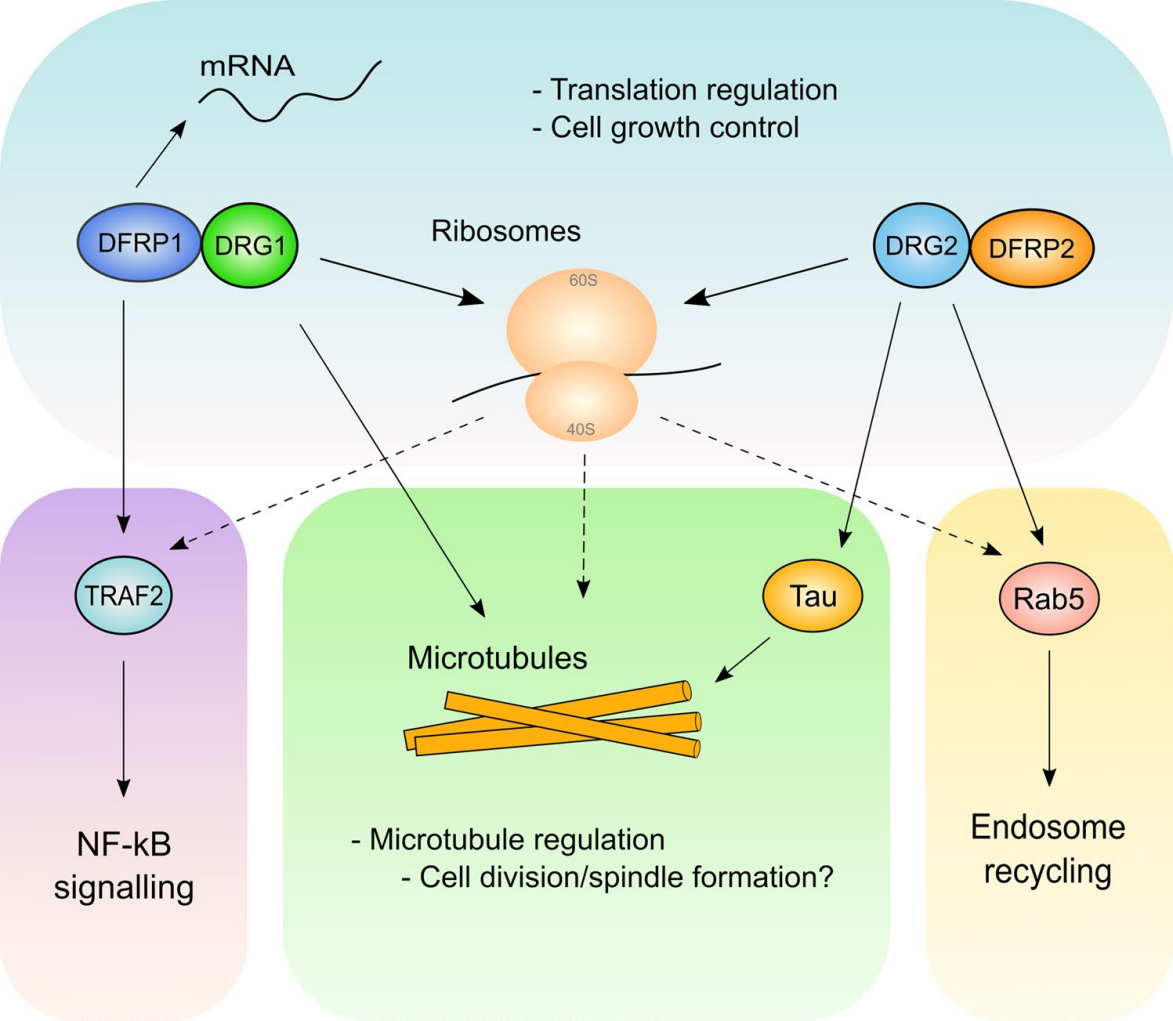
DRGs and DFRPs in mammalian cell biology. The DRG1/DFRP1 complex has been reported to interact with ribosomes in mammalian cells, suggesting its role in translational regulation may be conserved between orthologues. DFRP1 has also been reported to interact with TRAF2 and may have a role in NF-κβ signalling. Both DRGs have been suggested to regulate microtubules through either direct association as in the case of DRG1 or through regulation of Tau by DRG2. Whilst it has not been conclusively confirmed, mammalian DRG2 and DFRP2 also likely interact with and regulate ribosomes given the high sequence conservation with their yeast counterparts. Furthermore, DRG2 has also been implicated in the regulation of endosome recycling, via its interaction with Rab5. Given that DRGs and DFRPs are involved in translation, it is possible that deregulated protein synthesis could be contributing indirectly to the other reported functions. This potential regulation has been indicated using dashed arrows

In higher eukaryotes the role of DRGs and their DFRPs in translation is less clear, though given the high sequence conservation with their yeast counterparts, they likely have a similar function in translation elongation. Ishikawa et al. suggested that the mammalian DRG1/DFRP1 complex could associate with polysomes [32] (Fig. 6). However, a proteomic screen in mouse embryonic stem cells did not detect DRG1/DFRP1 as ribosome interactors [70]. DRG2 and DFRP2 were also not detected. This may suggest the DRG/DFRP complexes only interact with ribosomes under certain conditions, or could be due to technical differences. DFRP1 has, however, been identified in several proteomics screens as a mammalian mRNA binding protein, though DRG1 is not always present [71–73]. This is consistent with the zinc fingers of DFRP1 being predicted to interact with mRNA. However, more work is needed to understand which RNAs DFRP1 is capable of interacting with and whether or not it is limited to mRNA, ribosomal RNA, or some other RNA species. Furthermore, it should be noted that there is some evidence suggesting DRGs may bind RNA in isolation, as Ishikawa et al. reported *Xenopus* DRG1 and 2 could bind to poly uridine RNA in vitro [51]. DRG2 binding to poly cytidine RNA was also reported by Markolovic et al. who suggested the interaction might be regulated by hydroxylation of DRG2 [26] (see above). Interestingly, DRG1 and DFRP1 may also have a function outside of translation as they can both localize to the nucleus [26, 55, 56].

### DRGs and microtubules

Aside from their roles in translation, both DRG1 and DRG2 have also been implicated in the regulation of microtubules (Fig. 6). Microtubules are polymers of αβ-tubulin proteins and form an integral part of the cytoskeleton [74]. Furthermore, they are essential for proper cell division as they are responsible for chromosome segregation [74]. Lu et al. first suggested that DRG1 could co-localize with microtubules during mitosis and interact with proteins involved in the spindle assembly checkpoint such as BubR1 [56]. Overexpression of DRG1 was also suggested to increase the number of multinucleated cells and chromosomal lagging, suggesting that these cells had abnormal chromosome segregation due to deregulated microtubules and the spindle assembly checkpoint [56].

Schellhaus et al. subsequently reported that DRG1 and DFRP1, but not DFRP2 (DRG2 was not tested), could interact with microtubules in vitro [75]. Domain mapping experiments showed that DRG1 could partially interact with microtubules after deletion of both the TGS and HTH domains. However, on their own, both the TGS and HTH domains could interact strongly with microtubules [75]. Based on this evidence, the authors suggested that the GTPase domain, as well as the HTH and TGS domains, form one large interaction surface that promotes the bundling, polymerization and stabilization of microtubules in vitro. Interestingly, these proposed functions of DRG1 did not require its GTPase activity. In vivo, although spindles still formed during mitosis when DRG1 was depleted, there was a delay in the transition from prophase to anaphase, and from aster formation to the formation of the spindle. This suggests DRG1 may be required for optimal spindle dynamics during cell division [75]. It is not fully understood yet how DRG1 binds and regulates microtubules. Although DFRP1 was also shown to associate with microtubules, its role in DRG1’s association with and regulation of microtubules in vivo is not yet known.

As mentioned above, DRG2 has also been implicated in the regulation of microtubules (Fig. 6). Dang et al. proposed that knockdown of DRG2 in HeLa cells causes a decrease in fast growing microtubules but an increase in the amount of long-lived slow growing microtubules [76]. Microtubules were also more stable in DRG2-depleted HeLa cells after treatment with microtubule inhibitors such as paclitaxel [76]. DRG2 knockdown was also suggested to inhibit the formation of perinuclear microtubule organising centres and to cause Golgi fragmentation [76, 77]. This suggests DRG2 is promoting the polymerization of microtubules whilst in the absence of DRG2 the microtubules are stabilized. In addition to this, it has been suggested that overexpressing DRG2 in Jurkat T cells reduces sensitivity to the microtubule deregulating drug nocodazole [78]. This could be consistent with DRG2 promoting microtubule polymerization in nocodazole treated cells where microtubules may be depolymerized.

How DRGs might control microtubule dynamics is not yet clear. Interestingly however, DRG2 was shown to interact with Tau, a protein known to promote the stability of microtubules [76]. Furthermore, DRG2 knockdown decreases the phosphorylation of Tau which is thought to promote an interaction between Tau and microtubules, leading to increased microtubule stabilization [77]. Mani et al. suggested that DRG2 knockdown may impart this affect by causing an increase in the inhibitory phosphorylation of glycogen synthase kinase 3β (GSK3β) by Akt which then results in reduced phosphorylation of the GSK3β target protein Tau [77]. Therefore, DRG2 knockdown may prevent the depolymerization of microtubules by deregulating the activity of the Akt-GSK3β-Tau pathway. Although the mechanism by which DRG2 regulates Akt activity is unknown, Mani et al. suggested that an increase in epidermal growth factor receptor (EGFR) in endosomes caused by DRG2 depletion could lead to activation of Akt [77, 79].

### DRG1 and ciliogenesis

Interestingly, DRG1 has also been implicated in the regulation of cilia [80]. Cilia are long, thin, microtubule structures that protrude from the surface of cells where they are important for signalling pathways and development [81]. Lee et al. suggested that DRG1 promotes the formation of cilia independently of its GTPase activity, through an interaction with the Wnt signalling activator Dishevelled (Dvl) [80]. DRG1 and Dvl interact with the protein Daam1 (Dishevelled-associated activator of morphogenesis 1), which in turn promotes the formation of the apical actin network that is required for proper basal body localization and cilia formation [80]. Importantly, cilia are required for many crucial developmental signalling pathways such as Hedgehog (Hh), Wnt, Notch, Hippo, PDGFR and mTOR [82]. Whether or not DRG1 is required for these signalling cascades is unknown.

### DRG2: endosomal recycling and membrane tubules

As well as a role in microtubule regulation, DRG2 has also been implicated in the regulation of endosomes (Fig. 6). Mani et al. reported that DRG2 can co-localize with endosomes and interact with early endosomal proteins including Rab5, which is a small GTPase involved in the regulation of early endosome formation [79]. DRG2 localization to endosomes was dependent on PI3K signalling and may be dependent on binding endosomal proteins like Rab5 [79]. Furthermore, DRG2 knockdown was reported to cause a defect in endosomal recycling, as indicated by the retention of transferrin (Tfn) [79], a marker commonly used to indicate a defect in endosomal recycling. For proper endosomal recycling, the Rab5 GTPase must be inactivated by GAP proteins such as RabGAP5, which trigger Rab5 hydrolysis of GTP into GDP. Mani et al. suggested that DRG2 is required for RabGAP5 to interact with, and inactivate, Rab5 [79]. Without Rab5 deactivation, the endosomes are not recycled to the cell membrane, thus causing retention of Tfn. However, it is not yet known how DRG2 might regulate the interaction between Rab5 and RabGAP5.

Similar to its role in endosomes, DRG2 is also involved in the regulation of membrane tubules [83]. Membrane tubules are formed as part of intracellular organelles such as the Golgi and endoplasmic reticulum. DRG2 was shown to be capable of localizing to membrane tubules where it interacts with GTP bound Rac1 [83]. Depletion of DRG2 caused a decrease in the length of Rac1 membrane tubules suggesting DRG2 is required for the stabilization of these structures [83].

Taken together, these studies suggest that DRG2 may be involved in endosome recycling and membrane tubule stabilization; however, it is unknown whether DRG2 requires its GTPase activity or its binding partner DFRP2 for these functions.

### DRG2 and mitochondria

DRG2 has also been implicated in mitochondrial dynamics [84]. Depletion of DRG2 in HeLa cells was reported to cause mitochondrial swelling, which was accompanied by a loss of mitochondrial membrane potential and a decrease in the production of ATP. Vo et al. suggested that DRG2 knockdown causes a decrease in the mRNA and protein levels of the mitochondrial fission protein Drp1 (dynamin-related protein 1) [84]. Furthermore, Vo et al. reported that overexpressing Drp1 in DRG2-depleted cells rescued the mitochondrial dysfunction, suggesting DRG2 depletion causes mitochondrial deregulation via downregulating Drp1 [84]. How DRG2 downregulates the expression of Drp1 is unknown.

## Roles of DRGs and DFRPs in disease

### Cell proliferation and cancer

The DRG GTPases have been implicated in a variety of cellular processes including cell growth and proliferation. Several papers have suggested that DRG1, DRG2 and DFRP1 are required for the growth and proliferation of various cancer cell lines [56, 84–88]. Together, these reports suggest that DRG GTPases promote the growth and viability of proliferating cells. This is supported by expression analysis showing that these proteins are highly expressed during development and in actively growing tissues [50, 53] (see section on expression above).

Consistent with the DRG1/DFRP1 complex being required for cancer cell proliferation there is emerging evidence supporting an oncogenic role in cancer. As discussed in relation to microtubules, Lu et al. showed that knockdown of DRG1 in two lung cancer cell lines causes a decrease in cell proliferation and an increase in cells in G2/M [56], suggesting DRG1 is required for proper cell cycling. There is also evidence suggesting that DRG1 is overexpressed in lung adenocarcinoma [56]. In addition to this, DRG1 was found to interact with the protein TAL1 (T-cell acute lymphoblastic leukemia) and to stimulate the co-transforming ability of c-Myc and Ras in rat embryonic fibroblasts [89].

A recent report has shown evidence that DRG1 is overexpressed in osteosarcoma, where its expression is correlated with tumor size and clinical progression [87]. Ling et al. reported that DRG1 knockdown in two osteosarcoma cell lines causes reduced cell viability and colony formation as well as causing an increase in apoptosis and a G2/M arrest [87]. Furthermore, Ling et al. suggested that DRG1 expression in osteosarcoma is dependent on the N6-methyladenosine modification of its mRNA, as knockdown of the RNA methyltransferase METTL3 reduced DRG1 expression [87].

DRG1 is also overexpressed in melanoma, where knocking down its expression reduces cell proliferation and soft agar colony formation [90]. Interestingly, the authors of this study also identified DRG1 as a melanoma-associated antigen that is recognized by CD4 + T cells, raising the possibility that it could be a novel target for cancer immunotherapy [90].

DFRP1 is overexpressed in hepatocellular carcinomas where high expression is correlated with decreased survival [86]. In a hepatocellular cell line knockdown of DFRP1 results in reduced cell proliferation in addition to causing an increase in apoptosis [86]. Depleting DFRP1 also reduced the growth of tumors in mice [86].

DFRP1 is also overexpressed in acute myeloid leukaemia (AML) and interacts with TRAF2 (Fig. 6) [55, 86]. TRAF2 is an E3 ubiquitin ligase and is involved in NF-κB signalling and apoptosis. Interestingly, knocking down DFRP1 was suggested to increase apoptosis and decrease the transcriptional activity of NF-κB [86]. Furthermore, Jiang et al. also reported that DFRP1 knockdown decreased the phosphorylation of the p65 subunit of NF-κB [86]. The activity of NF-κB could be partially rescued in cells overexpressing TRAF2, suggesting DFRP1 is involved in TRAF2’s positive regulation of NF-κB [86]. It is unknown if DRG1 also interacts with TRAF2 and regulates NF-κB signalling with DFRP1.

Whilst DFRP1 has been suggested to have an oncogenic role, there have been no reports yet implicating DFRP2 in cancer. However, this might be inferred from growing evidence supporting a role of DRG2 in tumorigenesis. Depletion of DRG2 in HeLa cells results in reduced cell proliferation and an increase in apoptosis [84, 85]. Specifically, Jang et al. reported that DRG2 knockdown in HeLa cells causes a G2/M block [85], which is similar to knockdown of DRG1 in lung cancer and osteosarcoma cell lines, as noted above [56, 87]. DRG2 knockdown was reported to upregulate p21, Myt1 and Wee1 and inhibit the activity of the cyclin B1/Cdk1 complex leading to G2/M arrest [85]. In contrast to this, it has been reported that overexpressing DRG2 in Jurkat T cells reduces proliferation and increases cells in G2/M [78, 91]. This differing result may be due to the very different nature of T cells compared to HeLa cells or due to indirect effects caused by overexpression of DRG2.

Hong et al. suggested that DRG2 is overexpressed in some non-small cell lung cancers [92]. The increase in DRG2 expression may be due to an intronic variant which supposedly increases the promoter activity of the *DRG2* gene, despite the variant being present in a neighboring gene [92]. The manner in which this variant might affect the expression of DRG2 is unknown. DRG2 has also been implicated in melanoma [88]. Yoon et al. reported that DRG2 is overexpressed in some melanomas and that this expression shows positive correlation with metastatic spread and reduced patient survival [88]. Furthermore, depletion of DRG2 reduces the proliferation and soft agar colony formation of melanoma cells whilst also reducing tumor growth and metastasis in mice [88].

The research discussed here suggests that DRG/DFRP complexes support oncogenesis and thus may be potential therapeutic targets for cancer treatment. Oncogenic functions could be consistent with one or more of the cellular roles detailed above, including translation, cytoskeletal regulation, endocytosis and cell growth. Further work is required to pinpoint which functions are required for the roles of DRGs and DFRPs in cancer.

How might DRG/DFRP complexes be targeted? Disruption of the DRG-DFRP interaction might be a potential strategy, as it would result in the degradation of the DRG proteins. Other ways of targeting these pathways could be the use of GTPase inhibitors or inhibitors of the DRG hydroxylase JMJD7. Although small molecule inhibitors are under development for a variety of small GTPases and their GAPs/GEFs, this is recognized as a challenging area [93]. Interestingly, inhibitors have been successfully developed for HIF hydroxylases and some other 2OG oxygenases [45], suggesting that JMJD7 inhibition could also be a viable approach.

### Developmental disorders

Aside from their role in cancer, there have also been several reports indicating that DRGs are involved in some developmental/neurological disorders. Al-Nabhani et al. identified a homozygous truncation mutation (G54*) in *DRG1* in a patient with microcephaly and developmental delay [94]. *DRG1* is also a candidate gene for Autism spectrum disorders [95]. Interestingly, the gene encoding JMJD7 also contains reported mutations that are linked to Autism spectrum disorders [96]. Furthermore, the *DRG1* gene is located on chr22q12, a region that is duplicated in patients with intellectual difficulties and growth retardation [97, 98].

The *DRG2* gene is located in a region of chromosome 17 that is duplicated in patients with Smith–Magenis syndrome (SMS) and Potocki–Lupski syndrome [99, 100]. SMS causes intellectual disability, facial abnormalities and behavioral problems, whilst Potocki–Lupski can cause delayed development, autism and cardiovascular problems. Consistent with a role for the *DRG2* gene in brain development, Lim et al. reported that *DRG2* knockout mice had reduced motor function, likely as a result of deregulated dopamine neurons [101].

It is interesting to note that deregulation of translational control has previously been implicated in neurological disorders [102]. Thus, the involvement of DRGs in neurological disorders could be consistent with a role in translational control.

## Conclusions and future perspectives

Here we have reviewed the current understanding of both the DRG GTPases and their binding partners, DFRP1 and DFRP2. Both DRG/DFRP complexes appear to have important roles in the regulation of cell growth and translation consistent with the functions of related OBG GTPases. However, more recent work has significantly expanded the number of proposed DRG functions to include the regulation of microtubules, endosomes, mitochondria, etc. In the future it will be important to determine whether these represent direct and/or indirect effects. For example, deregulated translation could negatively impact the normal expression of many proteins, giving rise to a variety of cellular phenotypes. Alternatively, DRGs might regulate multiple independent effectors that control distinct cellular processes, either in parallel or in specific biological contexts. Determining which DRG interactions are GTP-dependent would likely support our understanding of their direct functions, and the molecular mechanisms involved.

Given the high sequence similarity between DRG orthologues in vertebrates and lower organisms such as yeast and Archaea, it is difficult to explain how the functions of DRGs have grown to include such diverse functions. One possibility is that the evolution of the DFRP proteins has allowed new functions for DRGs to evolve. As discussed in an earlier section, the DFRP proteins are much less conserved when compared to DRGs, but appear to have maintained strong binding specificity for their respective DRG. This could explain how the DRGs might have evolved new functions in vertebrates that maybe do not fit with the presence of highly similar DRG orthologues in yeast and Archaea.

Future work should investigate the determinants controlling specificity between DRG1 and DRG2, including the role of PTMs such as phosphorylation and hydroxylation, and interacting proteins including DFRPs and effectors. Specific attention should be applied to the role of DFRP proteins in the regulation of DRGs given that they are often overlooked. Considering the high level of sequence conservation between DRG1 and DRG2 it will be of interest to understand how specificity is achieved at the molecular level: such studies would be aided by structures of human DRG/DFRP complexes, particularly in complex with GTP analogues and effector proteins.

The high degree of sequence conservation between DRG1 and DRG2 may also present a number of technical challenges that are worth highlighting here for future consideration. For example, we have observed that a number of commercially available DRG antibodies cross-react with the other DRG protein, and that the amount of cross-reactivity is effected by the relative abundance of DRG1 and DRG2 in the cell type in question (QZ, personal communication); therefore, we would recommend that antibody specificity is proven on a case-by-case basis using, for example, RNA knockdown approaches.

Whether or not there is any functional redundancy between DRG1 and DRG2 should also be considered in future work. Furthermore, the effect of experimentally overexpressing these proteins on the binding specificity between DRGs and DFRPs is often not considered. Whether any change in binding specificity is biologically relevant is unclear, though it could be possible under conditions where the proteins are physiologically or pathologically overexpressed, such as during development or cancer.

Overall, we believe the high conservation of DRG/DFRP complexes and their links to fundamental cellular processes, as well as diseases such as cancer, make them an important and exciting topic of research for the future.

## Supplementary Information

Below is the link to the electronic supplementary material.

Supplementary file1 (PDF 179 KB)

## Data Availability

Not applicable.

## Abbreviations

DRG: Developmentally regulated GTP-binding protein
DFRP: DRG family regulatory protein
GTP/GDP: Guanosine tri/di-phosphate
Rbg: Ribosome binding GTPase

## References

[1] Li B, Trueb B (2000). DRG represents a family of two closely related GTP-binding proteins. Biochim Biophys Acta.

[2] Ishikawa K, Azuma S, Ikawa S, Semba K, Inoue J (2005). Identification of DRG family regulatory proteins (DFRPs): specific regulation of DRG1 and DRG2. Genes Cells.

[3] Daugeron MC, Prouteau M, Lacroute F, Seraphin B (2011). The highly conserved eukaryotic DRG factors are required for efficient translation in a manner redundant with the putative RNA helicase Slh1. Nucleic Acids Res.

[4] Leipe DD, Wolf YI, Koonin EV, Aravind L (2002). Classification and evolution of P-loop GTPases and related ATPases1. J Mol Biol.

[5] Wittinghofer A, Vetter IR (2011). Structure-function relationships of the G domain, a canonical switch motif. Annu Rev Biochem.

[6] Sahai E, Marshall CJ (2002). RHO–GTPases and cancer. Nat Rev Cancer.

[7] Wennerberg K, Rossman KL, Der CJ (2005). The Ras superfamily at a glance. J Cell Sci.

[8] Bourne HR, Sanders DA, McCormick F (1991). The GTPase superfamily: conserved structure and molecular mechanism. Nature.

[9] Vetter IR, Wittinghofer A (2001). The guanine nucleotide-binding switch in three dimensions. Science.

[10] Bos JL, Rehmann H, Wittinghofer A (2007). GEFs and GAPs: critical elements in the control of small G proteins. Cell.

[11] Fernández-Medarde A, Santos E (2011). Ras in cancer and developmental diseases. Genes Cancer.

[12] Kumar S, Tomooka Y, Noda M (1992). Identification of a set of genes with developmentally down-regulated expression in the mouse brain. Biochem Biophys Res Commun.

[13] Sazuka T, Tomooka Y, Ikawa Y, Noda M, Kumar S (1992). DRG: a novel developmentally regulated GTP-binding protein. Biochem Biophys Res Commun.

[14] Hudson JD, Young PG (1993). Sequence of the Schizosaccharomyces pombe gtp1 gene and identification of a novel family of putative GTP-binding proteins. Gene.

[15] Kumar S, Iwao M, Yamagishi T, Noda M, Asashima M (1993). Expression of GTP-binding protein gene drg during Xenopus laevis development. Int J Dev Biol.

[16] Schenker T, Lach C, Kessler B, Calderara S, Trueb B (1994). A novel GTP-binding protein which is selectively repressed in SV40 transformed fibroblasts. J Biol Chem.

[17] Caldon CE, Yoong P, March PE (2001). Evolution of a molecular switch: universal bacterial GTPases regulate ribosome function. Mol Microbiol.

[18] Kallstrom G, Hedges J, Johnson A (2003). The putative GTPases Nog1p and Lsg1p are required for 60S ribosomal subunit biogenesis and are localized to the nucleus and cytoplasm, respectively. Mol Cell Biol.

[19] Zhang S, Haldenwang WG (2004). Guanine nucleotides stabilize the binding of Bacillus subtilis Obg to ribosomes. Biochem Biophys Res Commun.

[20] Feng B, Mandava CS, Guo Q, Wang J, Cao W, Li N (2014). Structural and functional insights into the mode of action of a universally conserved Obg GTPase. PLoS Biol.

[21] Rudra P, Hurst-Hess KR, Cotten KL, Partida-Miranda A, Ghosh P (2020). Mycobacterial HflX is a ribosome splitting factor that mediates antibiotic resistance. Proc Natl Acad Sci USA.

[22] Gkekas S, Singh RK, Shkumatov AV, Messens J, Fauvart M, Verstraeten N (2017). Structural and biochemical analysis of *Escherichia**coli* ObgE, a central regulator of bacterial persistence. J Biol Chem.

[23] Buglino J, Shen V, Hakimian P, Lima CD (2002). Structural and biochemical analysis of the Obg GTP binding protein. Structure.

[24] Teplyakov A, Obmolova G, Chu SY, Toedt J, Eisenstein E, Howard AJ (2003). Crystal structure of the YchF protein reveals binding sites for GTP and nucleic acid. J Bacteriol.

[25] Francis SM, Gas ME, Daugeron MC, Bravo J, Seraphin B (2012). Rbg1-Tma46 dimer structure reveals new functional domains and their role in polysome recruitment. Nucleic Acids Res.

[26] Markolovic S, Zhuang Q, Wilkins SE, Eaton CD, Abboud MI, Katz MJ (2018). The Jumonji-C oxygenase JMJD7 catalyzes (3S)-lysyl hydroxylation of TRAFAC GTPases. Nat Chem Biol.

[27] Ramakrishnan V, Davies C, Gerchman SE, Golden BL, Hoffmann DW, Jaishree TN (1995). Structures of prokaryotic ribosomal proteins: implications for RNA binding and evolution. Biochem Cell Biol.

[28] Harlow LS, Kadziola A, Jensen KF, Larsen S (2004). Crystal structure of the phosphorolytic exoribonuclease RNase PH from *Bacillus**subtilis* and implications for its quaternary structure and tRNA binding. Protein Sci.

[29] Wolf YI, Aravind L, Grishin NV, Koonin EV (1999). Evolution of aminoacyl-tRNA synthetases—analysis of unique domain architectures and phylogenetic trees reveals a complex history of horizontal gene transfer events. Genome Res.

[30] Verstraeten N, Fauvart M, Versées W, Michiels J (2011). The universally conserved prokaryotic GTPases. Microbiol Mol Biol Rev.

[31] Zeng F, Li X, Pires-Alves M, Chen X, Hawk CW, Jin H (2021). Conserved heterodimeric GTPase Rbg1/Tma46 promotes efficient translation in eukaryotic cells. bioRxiv.

[32] Ishikawa K, Akiyama T, Ito K, Semba K, Inoue J (2009). Independent stabilizations of polysomal Drg1/Dfrp1 complex and non-polysomal Drg2/Dfrp2 complex in mammalian cells. Biochem Biophys Res Commun.

[33] Chen J, Shen B-Y, Deng X-X, Zhan Q, Peng C-H (2012). SKP1-CULLIN1-F-box (SCF)-mediated DRG2 degradation facilitated chemotherapeutic drugs induced apoptosis in hepatocellular carcinoma cells. Biochem Biophys Res Commun.

[34] Alves VS, Castilho BA (2005). Gir2 is an intrinsically unstructured protein that is present in Saccharomyces cerevisiae as a group of heterogeneously electrophoretic migrating forms. Biochem Biophys Res Commun.

[35] Doerks T, Copley RR, Schultz J, Ponting CP, Bork P (2002). Systematic identification of novel protein domain families associated with nuclear functions. Genome Res.

[36] Wout PK, Sattlegger E, Sullivan SM, Maddock JR (2009). Saccharomyces cerevisiae Rbg1 protein and its binding partner Gir2 interact on Polyribosomes with Gcn1. Eukaryot Cell.

[37] Ishikawa K, Ito K, Inoue J-I, Semba K (2013). Cell growth control by stable Rbg2/Gir2 complex formation under amino acid starvation. Genes Cells.

[38] O'Connell A, Robin G, Kobe B, Botella JR (2009). Biochemical characterization of Arabidopsis developmentally regulated G-proteins (DRGs). Protein Expr Purif.

[39] Perez-Arellano I, Spinola-Amilibia M, Bravo J (2013). Human Drg1 is a potassium-dependent GTPase enhanced by Lerepo4. FEBS J.

[40] Ash M-R, Maher MJ, Mitchell Guss J, Jormakka M (2012). The cation-dependent G-proteins: in a class of their own. FEBS Lett.

[41] Eswaran J, Bernad A, Ligos JM, Guinea B, Debreczeni JE, Sobott F (2008). Structure of the human protein kinase MPSK1 reveals an atypical activation loop architecture. Structure.

[42] Wu H, Sun L, Blombach F, Brouns SJJ, Snijders APL, Lorenzen K (2010). Structure of the ribosome associating GTPase HflX. Proteins.

[43] Koller-Eichhorn R, Marquardt T, Gail R, Wittinghofer A, Kostrewa D, Kutay U (2007). Human OLA1 defines an ATPase subfamily in the Obg family of GTP-binding proteins. J Biol Chem.

[44] Ploumakis A, Coleman ML (2015). OH, the places you’ll go! hydroxylation, gene expression, and cancer. Mol Cell.

[45] Islam MS, Leissing TM, Chowdhury R, Hopkinson RJ, Schofield CJ (2018). 2-oxoglutarate-dependent oxygenases. Annu Rev Biochem.

[46] Fletcher SC, Coleman ML (2020). Human 2-oxoglutarate-dependent oxygenases: nutrient sensors, stress responders, and disease mediators. Biochem Soc Trans.

[47] Schofield CJ, Ratcliffe PJ (2004). Oxygen sensing by HIF hydroxylases. Nat Rev Mol Cell Biol.

[48] Loenarz C, Schofield CJ (2011). Physiological and biochemical aspects of hydroxylations and demethylations catalyzed by human 2-oxoglutarate oxygenases. Trends Biochem Sci.

[49] Feng T, Yamamoto A, Wilkins SE, Sokolova E, Yates LA, Münzel M (2014). Optimal translational termination requires C4 lysyl hydroxylation of eRF1. Mol Cell.

[50] Sazuka T, Kinoshita M, Tomooka Y, Ikawa Y, Noda M, Kumar S (1992). Expression of DRG during murine embryonic development. Biochem Biophys Res Commun.

[51] Ishikawa K, Azuma S, Ikawa S, Morishita Y, Gohda J, Akiyama T (2003). Cloning and characterization of Xenopus laevis drg2, a member of the developmentally regulated GTP-binding protein subfamily. Gene.

[52] Devitt ML, Maas KJ, Stafstrom JP (1999). Characterization of DRGs, developmentally regulated GTP-binding proteins, from pea and Arabidopsis. Plant Mol Biol.

[53] Etheridge N, Trusov Y, Verbelen JP, Botella JR (1999). Characterization of ATDRG1, a member of a new class of GTP-binding proteins in plants. Plant Mol Biol.

[54] Lavdovskaia E, Kolander E, Steube E, Mai MM-Q, Urlaub H, Richter-Dennerlein R (2018). The human Obg protein GTPBP10 is involved in mitoribosomal biogenesis. Nucleic Acids Res.

[55] Capalbo G, Mueller-Kuller T, Koschmieder S, Klein H-U, Ottmann OG, Hoelzer D (2013). Characterization of ZC3H15 as a potential TRAF-2-interacting protein implicated in the NFκB pathway and overexpressed in AML. Int J Oncol.

[56] Lu L, Lv Y, Dong J, Hu S, Peng R (2016). DRG1 is a potential oncogene in lung adenocarcinoma and promotes tumor progression via spindle checkpoint signaling regulation. Oncotarget.

[57] Fleischer TC, Weaver CM, McAfee KJ, Jennings JL, Link AJ (2006). Systematic identification and functional screens of uncharacterized proteins associated with eukaryotic ribosomal complexes. Genes Dev.

[58] Hall TMT (2005). Multiple modes of RNA recognition by zinc finger proteins. Curr Opin Struct Biol.

[59] Beckmann BM, Horos R, Fischer B, Castello A, Eichelbaum K, Alleaume A-M (2015). The RNA-binding proteomes from yeast to man harbour conserved enigmRBPs. Nat Commun.

[60] Matia-González AM, Laing EE, Gerber AP (2015). Conserved mRNA-binding proteomes in eukaryotic organisms. Nat Struct Mol Biol.

[61] Mitchell SF, Jain S, She M, Parker R (2013). Global analysis of yeast mRNPs. Nat Struct Mol Biol.

[62] Marton MJ, Vazquez de Aldana CR, Qiu H, Chakraburtty K, Hinnebusch AG (1997). Evidence that GCN1 and GCN20, translational regulators of GCN4, function on elongating ribosomes in activation of eIF2alpha kinase GCN2. Mol Cell Biol.

[63] Kubota H, Sakaki Y, Ito T (2000). GI domain-mediated association of the eukaryotic initiation factor 2alpha kinase GCN2 with its activator GCN1 is required for general amino acid control in budding yeast. J Biol Chem.

[64] Pochopien AA, Beckert B, Kasvandik S, Berninghausen O, Beckmann R, Tenson T (2021). Structure of Gcn1 bound to stalled and colliding 80S ribosomes. Proc Natl Acad Sci USA.

[65] Juszkiewicz S, Speldewinde SH, Wan L, Svejstrup JQ, Hegde RS (2020). The ASC-1 complex disassembles collided ribosomes. Mol Cell.

[66] Searfoss A, Dever TE, Wickner R (2001). Linking the 3' poly(a) tail to the subunit joining step of translation initiation: relations of Pab1p, eukaryotic translation initiation factor 5B (Fun12p), and Ski2p-Slh1p. Mol Cell Biol.

[67] Dango S, Mosammaparast N, Sowa ME, Xiong L-J, Wu F, Park K (2011). DNA unwinding by ASCC3 helicase is coupled to ALKBH3-dependent DNA alkylation repair and cancer cell proliferation. Mol Cell.

[68] Williamson L, Saponaro M, Boeing S, East P, Mitter R, Kantidakis T (2017). UV irradiation induces a non-coding RNA that functionally opposes the protein encoded by the same gene. Cell.

[69] Matsuo Y, Ikeuchi K, Saeki Y, Iwasaki S, Schmidt C, Udagawa T (2017). Ubiquitination of stalled ribosome triggers ribosome-associated quality control. Nat Commun.

[70] Simsek D, Tiu GC, Flynn RA, Byeon GW, Leppek K, Xu AF (2017). The mammalian ribo-interactome reveals ribosome functional diversity and heterogeneity. Cell.

[71] Baltz AG, Munschauer M, Schwanhäusser B, Vasile A, Murakawa Y, Schueler M (2012). The mRNA-bound proteome and its global occupancy profile on protein-coding transcripts. Mol Cell.

[72] Castello A, Fischer B, Eichelbaum K, Horos R, Beckmann BM, Strein C (2012). Insights into RNA biology from an atlas of mammalian mRNA-binding proteins. Cell.

[73] Perez-Perri JI, Rogell B, Schwarzl T, Stein F, Zhou Y, Rettel M (2018). Discovery of RNA-binding proteins and characterization of their dynamic responses by enhanced RNA interactome capture. Nat Commun.

[74] Desai A, Mitchison TJ (1997). Microtubule polymerization dynamics. Annu Rev Cell Dev Biol.

[75] Schellhaus AK, Moreno-Andres D, Chugh M, Yokoyama H, Moschopoulou A, De S (2017). Developmentally regulated GTP binding protein 1 (DRG1) controls microtubule dynamics. Sci Rep.

[76] Dang T, Jang SH, Back SH, Park JW, Han I-S (2018). DRG2 deficiency causes impaired microtubule dynamics in HeLa cells. Mol Cells.

[77] Mani M, Thao DT, Kim BC, Lee UH, Kim DJ, Jang SH (2019). DRG2 knockdown induces Golgi fragmentation via GSK3β phosphorylation and microtubule stabilization. Biochim Biophys Acta Mol Cell Res.

[78] Song H, Kim S-I, Ko MS, Kim HJ, Heo JC, Lee HJ (2004). Overexpression of DRG2 increases G2/M phase cells and decreases sensitivity to nocodazole-induced apoptosis. J Biochem.

[79] Mani M, Lee UH, Yoon NA, Kim HJ, Ko MS, Seol W (2016). Developmentally regulated GTP-binding protein 2 coordinates Rab5 activity and transferrin recycling. Mol Biol Cell.

[80] Lee M, Hwang Y-S, Yoon J, Sun J, Harned A, Nagashima K (2019). Developmentally regulated GTP-binding protein 1 modulates ciliogenesis via an interaction with dishevelled. J Cell Biol.

[81] Goetz SC, Anderson KV (2010). The primary cilium: a signalling centre during vertebrate development. Nat Rev Genet.

[82] Wheway G, Nazlamova L, Hancock JT (2018). Signaling through the primary cilium. Front Cell Dev Biol.

[83] Mani M, Lee UH, Yoon NA, Yoon EH, Lee BJ, Cho WJ (2017). Developmentally regulated GTP-binding protein 2 is required for stabilization of Rac1-positive membrane tubules. Biochem Biophys Res Commun.

[84] Vo M-T, Ko MS, Lee UH, Yoon EH, Lee BJ, Cho WJ (2017). Developmentally regulated GTP-binding protein 2 depletion leads to mitochondrial dysfunction through downregulation of dynamin-related protein 1. Biochem Biophys Res Commun.

[85] Jang SH, Kim A-R, Park N-H, Park JW, Han I-S (2016). DRG2 regulates G2/M progression via the cyclin B1-Cdk1 complex. Mol Cells.

[86] Jiang B-G, Wan Z-H, Huang J, Li L-M, Liu H, Fu S-Y (2016). Elevated ZC3H15 increases HCC growth and predicts poor survival after surgical resection. Oncotarget.

[87] Ling Z, Chen L, Zhao J (2020) m6A-dependent up-regulation of DRG1 by METTL3 and ELAVL1 promotes growth, migration, and colony formation in osteosarcoma. Biosci Rep. 10.1042/BSR20200282 10.1042/BSR20200282 PMC7178206 32266933

[88] Yoon NA, Jung SJ, Choi SH, Ryu JH, Mani M, Lee UH (2019). DRG2 supports the growth of primary tumors and metastases of melanoma by enhancing VEGF-A expression. FEBS J.

[89] Mahajan MA, Park ST, Sun XH (1996). Association of a novel GTP binding protein, DRG, with TAL oncogenic proteins. Oncogene.

[90] Kiniwa Y, Li J, Wang M, Sun C, Lee JE, Wang R-F (2015). Identification of DRG-1 as a melanoma-associated antigen recognized by CD4(+) Th1 cells. PLoS ONE.

[91] Ko MS, Lee UH, Kim SI, Kim HJ, Park JJ, Cha SJ (2004). Overexpression of DRG2 suppresses the growth of Jurkat T cells but does not induce apoptosis. Arch Biochem Biophys.

[92] Hong MJ, Yoo SS, Choi JE, Kang H-G, Do SK, Lee JH (2018). Functional intronic variant of SLC5A10 affects DRG2 expression and survival outcomes of early-stage non-small-cell lung cancer. Cancer Sci.

[93] Prieto-Dominguez N, Parnell C, Teng Y (2019). Drugging the small GTPase pathways in cancer treatment: promises and challenges. Cells.

[94] Al-Nabhani M, Al-Rashdi S, Al-Murshedi F, Al-Kindi A, Al-Thihli K, Al-Saegh A (2018). Reanalysis of exome sequencing data of intellectual disability samples: yields and benefits. Clin Genet.

[95] de Krom M, Staal WG, Ophoff RA, Hendriks J, Buitelaar J, Franke B (2009). A common variant in DRD3 receptor is associated with autism spectrum disorder. Biol Psychiatry.

[96] Matsunami N, Hensel CH, Baird L, Stevens J, Otterud B, Leppert T (2014). Identification of rare DNA sequence variants in high-risk autism families and their prevalence in a large case/control population. Mol Autism.

[97] Abeliovich D, Maor E, Bashan N, Carmi R (1989). Duplication of distal 22q. Am J Med Genet.

[98] Prasher VP, Roberts E, Norman A, Butler AC, Krishnan VH, McMullan DJ (1995). Partial trisomy 22 (q11.2-q13.1) as a result of duplication and pericentric inversion. J Med Genet.

[99] Shuib S, Saaid NN, Zakaria Z, Ismail J, Abdul Latiff Z (2017). Duplication 17p11.2 (potocki-lupski syndrome) in a child with developmental delay. Malays J Pathol.

[100] Vlangos CN, Das P, Patel PI, Elsea SH (2000). Assignment of developmentally regulated GTP-binding protein (DRG2) to human chromosome band 17p11.2 with somatic cell hybrids and localization to the Smith-Magenis syndrome critical interval. Cytogenet Cell Genet.

[101] Lim HR, Vo M-T, Kim DJ, Lee UH, Yoon JH, Kim H-J (2019). DRG2 deficient mice exhibit impaired motor behaviors with reduced striatal dopamine release. Int J Mol Sci.

[102] Costa-Mattioli M, Sossin WS, Klann E, Sonenberg N (2009). Translational control of long-lasting synaptic plasticity and memory. Neuron.

