# Supplementary material for: Developmentally regulated GTPases: structure, function and roles in disease
Source: Cell Mol Life Sci. 2021 Oct 19;78(23):7219–35. doi: 10.1007/s00018-021-03961-0 (PMC8629797; doi:10.1007/s00018-021-03961-0)
Supplement: Supplementary file 1 — Supplementary file1 (PDF 179 KB) [file 18_2021_3961_MOESM1_ESM.pdf]

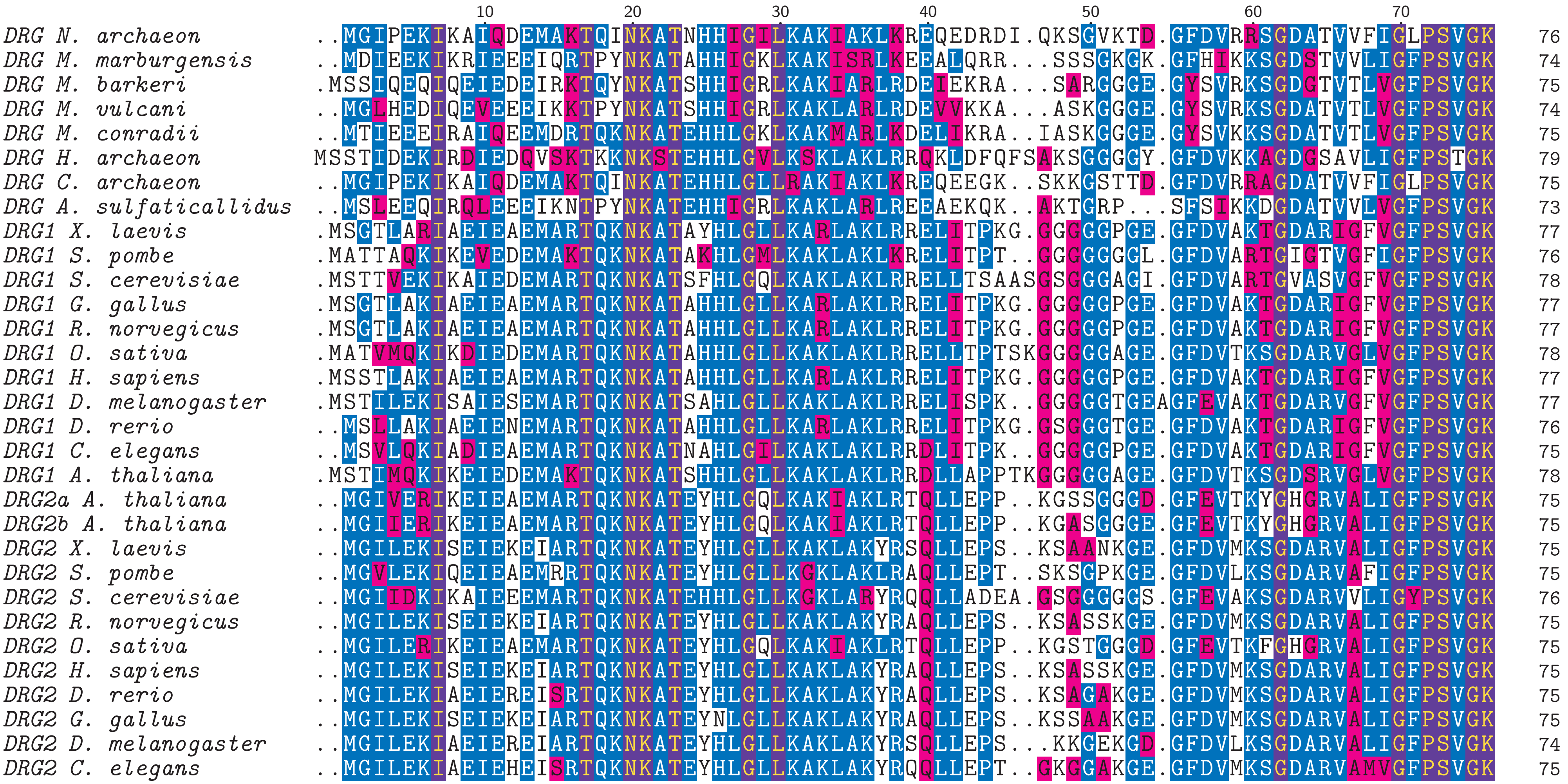

10 20 30 40 50 60 70
DRG N. archaeon ..MGIPEKIKAIQDEMAKTQINKATNHHIGILKAKIAKLKREQEDRDI.QKSGVKTD.GFDVRRSGDATVVFIGLPSVGK 76
DRG M. marburgensis ..MDIEEKIKRIEEEIQRTPYNKATAHHIGKLKAKISRLKEEALQRR...SSSGKGK.GFHIKKSGDSTVVLIGFPSVGK 74
DRG M. barkeri .MSSIQEQIQEIEDEIRKTQYNKATSHHIGRLKAKIARLRDEIEKRA...SARGGGE.GYSVRKSGDGTVTLVGFPSVGK 75
DRG M. vulcani ..MGLHEDIQEVEEEIKKTPYNKATSHHIGRLKAKLARLRDEVVKKA...ASKGGGE.GYSVRKSGDATVTLVGFPSVGK 74
DRG M. conradii ..MTIEEEIRAIQEEMDRTQKNKATEHHLGKLKAKMARLKDELIKRA..IASKGGGE.GYSVKKSGDATVTLVGFPSVGK 75
DRG H. archaeon MSSTIDEKIRDIEDQVSKTKKNKSTEHHLGVLKSKLAKLRRQKLDFQFSAKSGGGGY.GFDVKKAGDGSAVLIGFPSTGK 79
DRG C. archaeon ..MGIPEKIKAIQDEMAKTQINKATEHHLGLLRAKIAKLKREQEEGK..SKKGSTTD.GFDVRRAGDATVVFIGLPSVGK 75
DRG A. sulfaticallidus ..MSLEEQIRQLEEEIKNTPYNKATEHHIGRLKAKLARLREEAEKQK..AKTGRP...SFSIKKDGDATVVLVGFPSVGK 73
DRG1 X. laevis .MSGTLARIAEIEAEMARTQKNKATAYHLGLLKARLAKLRRELITPKG.GGGGPGE.GFDVAKTGDARIGFVGFPSVGK 77
DRG1 S. pombe .MATTAQKIKEVEDEMAKTQKNKATAKHLGMLKAKLAKLKRELITPT..GGGGGGL.GFDVARTGIGTVGFIGFPSVGK 76
DRG1 S. cerevisiae .MSTTVEKIKAIEDEMARTQKNKATSFHLGQLKAKLAKLRRELLTSAASGSGGGAGI.GFDVARTGVASVGFVGFPSVGK 78
DRG1 G. gallus .MSGTLAKIAEIEAEMARTQKNKATAHHLGLLKARLAKLRRELITPKG.GGGGPGE.GFDVAKTGDARIGFVGFPSVGK 77
DRG1 R. norvegicus .MSGTLAKIAEIEAEMARTQKNKATAHHLGLLKARLAKLRRELITPKG.GGGGPGE.GFDVAKTGDARIGFVGFPSVGK 77
DRG1 O. sativa .MATVMQKIKDIEDEMARTQKNKATAHHLGLLKAKLAKLRRELLTPTSKGGGGAGE.GFDVTKSGDARVGLVGFPSVGK 78
DRG1 H. sapiens .MSSTLAKIAEIEAEMARTQKNKATAHHLGLLKARLAKLRRELITPKG.GGGGPGE.GFDVAKTGDARIGFVGFPSVGK 77
DRG1 D. melanogaster .MSTILEKISAIESEMARTQKNKATSAHLGLLKAKLAKLRRELISPK..GGGGTGEAGFEVAKTGDARVGFVGFPSVGK 77
DRG1 D. rerio ..MSLLAKIAEIENEMARTQKNKATAHHLGLLKARLAKLRRELITPKG.GSGGTGE.GFDVAKTGDARIGFVGFPSVGK 76
DRG1 C. elegans ..MSVLQKIADIEAEMARTQKNKATNAHLGILKAKLAKLRRDLITPK..GGGGPGE.GFDVAKTGDARIGFVGFPSVGK 75
DRG1 A. thaliana .MSTIMQKIKEIEDEMAKTQKNKATSHHLGLLKAKLAKLRRDLLAPPTKGGGGAGE.GFDVTKSGDSRVGLVGFPSVGK 78
DRG2a A. thaliana ..MGIVERIKEIEAEMARTQKNKATEYHLGQLKAKIAKLRTQLLEPP..KGSSGGGD.GFEVTKYGHGRVALIGFPSVGK 75
DRG2b A. thaliana ..MGIIERIKEIEAEMARTQKNKATEYHLGQLKAKIAKLRTQLLEPP..KGASGGGE.GFEVTKYGHGRVALIGFPSVGK 75
DRG2 X. laevis ..MGILEKISEIEKEIARTQKNKATEYHLGLLKAKLAKYRSQLLEPS..KSAANKGE.GFDVMKSGDARVALIGFPSVGK 75
DRG2 S. pombe ..MGVLEKIQEIEAEMRRTQKNKATEYHLGLLKGKLAKLRAQLLEPT..SKSGPKGE.GFDVLKSGDARVAFIGFPSVGK 75
DRG2 S. cerevisiae ..MGIIDKIKAIEEEMARTQKNKATEHHLGLLKGKLARYRQQLLADEA.GSGGGGS.GFEVAKSGDARVVLIGYPSVGK 76
DRG2 R. norvegicus ..MGILEKISEIEKEIARTQKNKATEYHLGLLKAKLAKYRAQLLEPS..KSASSKGE.GFDVMKSGDARVALIGFPSVGK 75
DRG2 O. sativa ..MGILERIKEIEAEMARTQKNKATEYHLGQLKAKIAKLRTQLLEPP..KGSTGGGD.GFEVTKFGHGRVALIGFPSVGK 75
DRG2 H. sapiens ..MGILEKISEIEKEIARTQKNKATEYHLGLLKAKLAKYRAQLLEPS..KSASSKGE.GFDVMKSGDARVALIGFPSVGK 75
DRG2 D. rerio ..MGILEKIAEIEREISRTQKNKATEYHLGLLKAKLAKYRAQLLEPS..KSAGAKGE.GFDVMKSGDARVALIGFPSVGK 75
DRG2 G. gallus ..MGILEKISEIEKEIARTQKNKATEYNLGLLKAKLAKYRAQLLEPS..KSSAAKGE.GFDVMKSGDARVALIGFPSVGK 75
DRG2 D. melanogaster ..MGILEKIAEIEREIARTQKNKATEYHLGLLKAKLAKYRSQLLEPS...KKGEKGD.GFDVLKSGDARVALIGFPSVGK 74
DRG2 C. elegans ..MGILEKIAEIEHEISRTQKNKATEYHLGLLKAKLAKYRQQLLEPT..GKGGAKGE.GFDVMKSGDARVAMVGFPSVGK 75

```
                              80        90       100       110       120       130       140       150
DRG N. archaeon         STLLNKLTDAKSAVGAYQFTTLTVVPGMMNYRGAKIQVLDLPGIIKGASTGKGLGKRILSVARTADLVLLVLDVFQPF.H  155
DRG M. marburgensis     STLLNELTNAESKVGEYQFTTLEIVPGVMEYRGAQIQIFDIPGIITGASRGKGRGREILSVARSADLIVIVLDVFNTD.H  153
DRG M. barkeri          STLLNKITGANSAVGAYEFTTLTVVPGVLEHKGATIQFLDVPGLVKGASSGRGRGREVISVIRNSDMVIFLLDVFQPK.H  154
DRG M. vulcani          STLLNKLTGANSEVGAYEFTTLDVIPGVLEYNNATIQILDVPGLVKGAASGRGRGREVISVVRNCDLVVFILDVFQNY.H  153
DRG M. conradii         STLINKLTDAHSEVGEYEFTTLDVIPGIMEYRQAKIQILDLPGLVKGASAGRGRGREVISVIRSCDLIVMIIDVFNYQ.H  154
DRG H. archaeon         STLISSITSKESKIGHYAFTTTSAIPGIMFHKGTQIQIIDLPGIIEEASMGKGRGKQILAVARGADMIIIMIEPKKSTQY  159
DRG C. archaeon         STLLNRITGAKSAVGAFQFTTLTVVPGMMEYKGARIQVLDLPGIIKGASSGKGLGKRILSVARNADLVLLVLDVFQPY.H  154
DRG A. sulfaticallidus  SSLLNALTGAKSEIGAYDFTTLKPVPGMLEYNGAKIQIIDLPGIVEGASKGRGKGREIISAVRNSDLVVIIADPFNLE.S  152
DRG1 X. laevis          STLLSNLAGVYSEVAAYEFTTLTTVPGVVRYKGAKIQLLDLPGIIEGAKDGKGRGRQVIAVARTCNLILIVLDVLKPLGH  157
DRG1 S. pombe           STLMTQLTGTRSEAAAYEFTTLTTVPGVLQYNGAKIQILDLPGIIEGAKDGRGRGKQVITVARTCNLIFIVLDVLKPMSH  156
DRG1 S. cerevisiae      STLLSKLTGTESEAAEYEFTTLVTVPGVIRYKGAKIQMLDLPGIIDGAKDGRGRGKQVIAVARTCNLLFIILDVNKPLHH  158
DRG1 G. gallus          STLLSNLAGVYSEVAAYEFTTLTTVPGVIRYKGAKIQLLDLPGIIEGAKDGKGRGRQVIAVARTCNLILIVLDVLKPLGH  157
DRG1 R. norvegicus      STLLSNLAGVYSEVAAYEFTTLTTVPGVIRYKGAKIQLLDLPGIIEGAKDGKGRGRQVIAVARTCNLILIVLDVLKPLGH  157
DRG1 O. sativa          STLLNKLTGTFSEVAAYEFTTLTCIPGVIMYKGAKIQLLDLPGIIEGAKDGKGRGRQVISTARTCNVILIVLDAIKPITH  158
DRG1 H. sapiens         STLLSNLAGVYSEAAAYEFTTLTTVPGVIRYKGAKIQLLDLPGIIEGAKDGKGRGRQVIAVARTCNLILIVLDVLKPLGH  157
DRG1 D. melanogaster    STLLSNLAGVYSEVAAYEFTTLTTVPGCIKYKGAKIQLLDLPGIIEGAKDGKGRGRQVIAVARTCNLIFMVLDCLKPLGH  157
DRG1 D. rerio           STLLSNLAGVYSEVAAYEFTTLTTVPGVIRYKGAKIQLLDLPGIIEGAKDGKGRGRQVIAVARTCNLILIVLDVLKPLGH  156
DRG1 C. elegans         STLLCNLAGVFSEVAAYEFTTLTTVPGVIRYKGAKIQLLDLPGIIEGAKDGKGRGKQVIAVARTCSLILMVLDVMKPLQH  155
DRG1 A. thaliana        STLLNKLTGTFSEVASYEFTTLTCIPGVITYRGAKIQLLDLPGIIEGAKDGKGRGRQVISTARTCNCILIVLDAIKPITH  158
DRG2a A. thaliana       STLLTMLTGTHSEAASYEFTTLTCIPGVIHYNDTKIQLLDLPGIIEGASEGKGRGRQVIAVAKSSDLVLMVLDASKSEGH  155
DRG2b A. thaliana       STLLTMLTGTHSEAASYEFTTLTCIPGVIHYNDTKIQLLDLPGIIEGASEGKGRGRQVIAVAKSSDLVLMVLDASKSEGH  155
DRG2 X. laevis          STFLSLMTSTASEAASYEFTTLTCIPGVIEYKGANIQLLDLPGIIEGASQGKGRGRQVIAVARTSDVVIMMLDATKGEVQ  155
DRG2 S. pombe           STLLSAITKTKSATASYEFTTLTAIPGVLEYDGAEIQMLDLPGIIEGASQGRG.GRQAVSAARTADLILMVLDATKAADQ  154
DRG2 S. cerevisiae      SSLLGKITTTKSEIAHYAFTTLTSVPGVLKYQGAEIQIVDLPGIIYGASQGKGRGRQVVATARTADLVLMVLDATKSEHQ  156
DRG2 R. norvegicus      STFLSLMTSTASEAASYEFTTLTCIPGVIEYKGANIQLLDLPGIIEGAAQGRGRGRQVIAVARTADVVVMMLDATKGDVQ  155
DRG2 O. sativa          STLLTMLTGTHSEAASYEFTTLTCIPGIIQYNDTKIQLLDLPGIIEGASEGKGRGRQVIAVAKSSDLVLMVLDASKSEGH  155
DRG2 H. sapiens         STFLSLMTSTASEAASYEFTTLTCIPGVIEYKGANIQLLDLPGIIEGAAQGKGRGRQVIAVARTADVIIMMLDATKGEVQ  155
DRG2 D. rerio           STFLSLMTKTESEAASYEFTTLTCIPGVIEYKGANIQLLDLPGIIEGAAQGKGRGRQVIAVARTADVVIMMLDATKGDVQ  155
DRG2 G. gallus          STFLSLMTSTASEAASYEFTTLTCIPGVIEYKGANIQLLDLPGIIEGAAQGKGRGRQVIAVARTADVVIMMLDATKGEVQ  155
DRG2 D. melanogaster    STMLSTLTKTESEAANYEFTTLTCIPGVIEYQGANIQLLDLPGIIEGAAQGKGRGRQVIAVARTADLVLMMLDATKPNVH  154
DRG2 C. elegans         STLLSSMTSTHSEAAGYEFTTLTCIPGVISYNGANIQLLDLPGIIEGASQGKGRGRQVISVAKTADLILMMLDAGKSDQQ  155
```

```
                          160       170       180       190        200       210       220        230
DRG N. archaeon           EDVLVNELGSIGIRLNRLPPNITIEKSPMGGIAVAQQVKL.TKISEQHLKDILHIYGIVSARVVIRED.ITSEQLADHI.   232
DRG M. marburgensis       MNVILRELRDVGIRPNETPPDVTVKRRKLGGVKLSSTVEL.THLDERIIRSVLNEYGIHNADVLIRED.ITVDQFIDVM.   230
DRG M. barkeri            YEVLMDELYQAGIRIDQEPPDVVIKKNERGGIEINSTVDL..DLDEETIKAVLDEYKIHNASVLIRDN.ITVDQLIDVV.   230
DRG M. vulcani            HEVLTQELYDAGIRLNQKSPDVVIKRQDRGGININSTLDL..EISDDLIKAVLNDYKIHNAHVLIRDH.INVDQLIDAV.   229
DRG M. conradii           LKVLEDELYDAGIRINQRPPDVTITKLVKGGITITSTVEL.S.LDHETIKTILGEYKIHNALVNIRED.ITADQLIDVI.   230
DRG H. archaeon           LDKVLNELRKVAIRPGLKQPYIQIKKKDRGGIALSTLAKL.THMSEKTFVGILREYKIMNAAVTVRSD.PTIDELIDVL.   236
DRG C. archaeon           EDVLVNELGNIGIRLNQDPPNIVVEKTHTGGIAVAQQIKL.TKMSEKLLKDILNVYGMTSARVVIRED.ITSEQLIDFI.   231
DRG A. sulfaticallidus    IKIIQRELYNGGIRLNQKPPEVYVKKLERGGLKITSTVPL.S.IDEQTIYEVLREYRIHNAEVLIRED.VTVDRFIDAI.   228
DRG1 X. laevis            KKIIENELEGFGIRLNKQPPNIGFKKKDKGGINLTATCAQ.SELDNDTVKSILAEYKIHNADITLRSD.ATADDLIDVV.   234
DRG1 S. pombe             KRIIEEELEGFGIRLNKEPPNIVFKKKERGGINITNTVPL.THIDLDEIRAVCSEYRVNSADIAFRCD.ATIDDLIDVL.   233
DRG1 S. cerevisiae        KQIIEKELEGVGIRLNKTPPDILIKKKEKGGISITNTVPM.THLRNDEIRAVMSEYRINSAEIAFRCD.ATVDDLIDVL.   235
DRG1 G. gallus            KKIIENELEGFGIRLNSKPPNIGFKKKDKGGINLTATCPQ.SELDAETVKSILAEYKIHNADVTLRSD.ATADDLIDVV.   234
DRG1 R. norvegicus        KKIIENELEGFGIRLNSKPPNIGFKKKDKGGINLTATCPQ.SELDAETVKSILAEYKIHNADVTLRSD.ATADDLIDVV.   234
DRG1 O. sativa            KRLIEKELEGFGIRLNKTPPNLTFRRKDKGGINFTSTVTN.TNLDLETVKAICSEYRIHNADVSLRYD.ATADDLIDVI.   235
DRG1 H. sapiens           KKIIENELEGFGIRLNSKPPNIGFKKKDKGGINLTATCPQ.SELDAETVKSILAEYKIHNADVTLRSD.ATADDLIDVV.   234
DRG1 D. melanogaster      KKLLEHELEGFGIRLNKKPPNIYYKRKDKGGINLNSMVPQ.SELDTDLVKTILSEYKIHNADITLRYD.ATSDDLIDVI.   234
DRG1 D. rerio            KKLIEHELEGFGIRLNKQPPNIGFKKKDKGGINFTATCAQ.SELDGDTVKSILSEYKIHNADITLRSD.ATADDLIDVV.   233
DRG1 C. elegans          KKLLEYELEGFGIRLNKQPPNIGYKRKEKGGINLTMLVPQ.SELDLDLVKSILAEYRIHNADITLRYD.ATSEDLIDVI.   232
DRG1 A. thaliana         KRLIEKELEGFGIRLNKEPPNLTFRKKDKGGINLTSTVAV.THLDLDTVKAICGEYRMHNADITLRYD.ATADDLIDVI.   235
DRG2a A. thaliana        RQILTKELEAVGLRLNKRPPQIYFKKKKTGGISFNTTTPL.TRIDEKLCYQILHEYKIHNAEVLFRED.ATVDDFIDVV.   232
DRG2b A. thaliana        RQILTKELEAVGLRLNKTPPQIYFKKKKTGGISFNTTAPL.THIDEKLCYQILHEYKIHNAEVLFREN.ATVDDFIDVI.   232
DRG2 X. laevis            RSLLEMELESVGIRLNKRKPNIYFKPKKGGGISFNSTVPL.TQCSEKLVQLILHEYKMFNAEVLFRED.CTPDEFIDVI.   232
DRG2 S. pombe             REKIEYELEQVGIRLNRQPPNVTLTIKKNGGIKFNHTVPL.THMDYKMAYNILHEYRIHNADILIRED.ITVDDFIDLV.   231
DRG2 S. cerevisiae        RASLEKELENVGIRLNKEKPNIYYKKKETGGVKVTFTSPPKTNLTEQAIKMILRDYRIHNAEVLVRDDQCTIDDFIDVIN   236
DRG2 R. norvegicus        RSLLEKELESVGIRLNKHKPNIYFKPKKGGGISFNSTVTL.TQCSEKLVQLILHEYKIFNAEVLFRED.CSPDDFIDVI.   232
DRG2 O. sativa            RQILTRELEAVGLRLNKRPPQIYFKKKKTGGISFNSMIPL.THVDEKLCYQILHEYKIHNAEVLFRED.ATVDDFIDVI.   232
DRG2 H. sapiens           RSLLEKELESVGIRLNKHKPNIYFKPKKGGGISFNSTVTL.TQCSEKLVQLILHEYKIFNAEVLFRED.CSPDEFIDVI.   232
DRG2 D. rerio            RELLEKELESVGIRLNRPKPNIYFKPKKGGGLSYNSTVPL.TQCSEKLVQLILHEYKIFNAEVLFRED.CSPDDFIDVI.   232
DRG2 G. gallus           RALLEKELESVGIRLNKSKPNIYFKPKKGGGISFNSTVTL.TQCSEKLVQLILHEYKIFNAEVLFRED.CSPDEFIDVI.   232
DRG2 D. melanogaster      RELLERELESVGIRLNKRKPNIYFKQKKGGGLSFNATCSL.TRCNEKMVQTILHSFKIFNAEVLFRED.CTEDEFIDVV.   231
DRG2 C. elegans          KMLLERELEAVGIRLNKKPPNIYVKQKKVGGVKFTNTVPL.THCNEKLIMTVLHEYKIFNADVIFRED.CTVDEFIDVI.   232
```

```
                                    240       250       260       270       280       290       300
DRG N. archaeon            AG.NISYSKSLTILNKIDLVDREFLEDLKTKIKSDVIEVSANSDINIELLKEKIYEKLNFIRIYMRPKGGETDF..KEPL   309
DRG M. marburgensis        EA.NRAYIPAITVINKIDLVDESYLNHIKHEFP.DALLISADRKLNIDGLREEIFNRLGLIRIYMKPQGQKADY..SEPL   306
DRG M. barkeri             LN.NRSYVRSLIAVNKVDLAYPQLIEECRKLYP.NAIFISAHEGINIETLKDAIYDRLGFIRVYLKPQGQPADM..EEPL   306
DRG M. vulcani             MG.NRVYIPAVTVVNKVDMADEYVLKKCKAEYP.EATYISADKEVNLDSVKDLIYDALDFIRIYLKPQGGPADM..EEPL   305
DRG M. conradii            RG.NRVYIPAITVVNKIDLVDKEQLKLF....PKDALKISADMELNLDALREEIYNKLGFINIYLKPQGGPADM..DEPL   303
DRG H. archaeon            EG.NRVYPKLLILVNKIDLLSKKQRKRIDENIQ.DVLMISALTGENIENLKDEIVERLELIKIYLKKQREKTDF..EEPL   312
DRG C. archaeon            SG.SKTYSKALTIINKIDLVDETFLKELRTKIKSDFIEVSADSNVNIDLLKERIYEKLRFIRIYMRPKGGETDF..KEPL   308
DRG A. sulfaticallidus     LR.NRVYIPAIVVVNKIDLYNPGNL.......PDSVIPVSAEKKINLDLLAKSIYEKLDLIRIFLKPPGGKADL..NEPM   298
DRG1 X. laevis             EG.NRVYIPCIYVLNKIDQISIEELDII.YKVP.HCVPISAHHRWNFDDLLEKIWDYLQLVRIYTKPKGQLPDY..TSPV   309
DRG1 S. pombe              EG.NRVYIPALYVLNKIDSISIEELDLI.DRIP.NAVPICGNRGWNIDELKETMWDYLNLVRVYTRPRGLEPDY..SEPV   308
DRG1 S. cerevisiae         EASSRRYMPAVYVLNKIDSLSIEELELL.YRIP.NAVPISSGQDWNLDELLQVMWDRLNLVRIYTKPKGQIPDF..TDPV   311
DRG1 G. gallus             EG.NRVYIPCIYVLNKIDQISIEELDII.YKVP.HCVPISAHHRWNFDDLLEKIWDYLKLVRIYTKPKGQLPDY..TSPV   309
DRG1 R. norvegicus         EG.NRVYIPCIYVLNKIDQISIEELDII.YKVP.HCVPISAHHRWNFDDLLEKIWDYLKLVRIYTKPKGQLPDY..TSPV   309
DRG1 O. sativa             EG.SRIYMPCIYVVNKIDQITLEELEIL.DKLP.HYCPISAHLEWNLDGLLEMIWEYLDLVRIYTKPKGLNPDY..EDPV   310
DRG1 H. sapiens            EG.NRVYIPCIYVLNKIDQISIEELDII.YKVP.HCVPISAHHRWNFDDLLEKIWDYLKLVRIYTKPKGQLPDY..TSPV   309
DRG1 D. melanogaster       EG.NRIYIPCIYLLNKIDQISIEELDVI.YKIP.HCVPISAHHHWNFDDLLELMWEYLRLQRIYTKPKGQLPDY..NSPV   309
DRG1 D. rerio             EG.NRVYIPCIYVLNKIDQISIEELDVI.YKIP.HCVPISAHHRWNFDDLLEKIWDYLQLVRIYTKPKGQLPDY..TAPV   308
DRG1 C. elegans           EG.NRIYIPCIYVLNKIDQISIEELDII.YRIP.HTVPISAHHKWNFDDLLEKVWEYLNLIRIYTKPKGQLPDY..SQPI   307
DRG1 A. thaliana          EG.SRIYMPCIYAVNKIDSITLEELEIL.DKLP.HYCPVSAHLEWNLDGLLDKIWEYLDLTRIYTKPKAMNPDY..DDPV   310
DRG2a A. thaliana          EG.NRKYIKCVYVYNKIDVVGIDDVDRL.ARQP.NSIVISCNLKLNLDRLLARMWDEMGLVRVYSKPQSQQPDF..DEPF   307
DRG2b A. thaliana          EG.NRKYIKCVYVYNKIDVVGIDDVDRL.SRQP.NSIVISCNLKLNLDRLLARMWDEMGLVRVYSKPQGQQPDF..DEPF   307
DRG2 X. laevis             VG.NRVYMPCLYVYNKIDQISMEEVDRL.ARQP.YSVVISCGMKLNLDYLLEMLWENLALTCIYTKKRGERPDF..GDAI   307
DRG2 S. pombe              MG.NRRYINCLYCYSKIDAVSLEEVDRL.ARLP.KSVVISCNMKLNLDFLKERIWEELNLYRIYTKRKGEMPDF..SEAL   306
DRG2 S. cerevisiae         EQ.HRNYVKCLYVYNKIDAVSLEEVDKL.AREP.NTVVMSCEMDLGLQDVIEEIWYQLNLSRVYTKKRGVRPVF..DDPL   311
DRG2 R. norvegicus         VG.NRVYMPCLYVYNKIDQISMEEVDRL.ARKP.NSVVISCGMKLNLDYLLEMLWEYLALTCIYTKKRGQRPDF..TDAI   307
DRG2 O. sativa             EG.NRKYIKCVYVYNKIDVVGIDDVDKL.ARQP.NSLVISCNLQLNLDRLLARMWEEMGLVRVYTKPQGQQPDF..TDPV   307
DRG2 H. sapiens            VG.NRVYMPCLYVYNKIDQISMEEVDRL.ARKP.NSVVISCGMKLNLDYLLEMLWEYLALTCIYTKKRGQRPDF..TDAI   307
DRG2 D. rerio              VG.NRVYMPCLYVYNKVDQISIEEVDRL.AHRP.NSVVISCGMKLNLDYLLEQLWEYLALICIYTKKRGERPDF..GDPI   307
DRG2 G. gallus             VG.NRVYMPCLYVYNKIDQISMEEVDRL.ARRP.HSVVISCGMKLNLDYLLEKLWEYLALTCIYTKKRGQRPDF..TDAI   307
DRG2 D. melanogaster       TA.NRVYLPCLYVYNKIDQISIEEVDRL.ARQP.NSIVVSCNMKLNLDYMMEALWEALQLIRVYTKKPGAPPDF..DDGL   306
DRG2 C. elegans           QG.NRVYMTCLYVYNKVDQISIEEIDRL.ARMP.HHVVISCEMNLNMDYLLEKMWEYLALVRVYTKKPGNAPDLGPEDGI   309
```

```
                          310       320       330       340       350       360
DRG N. archaeon           IA...REGDSVEDICNKLHRSMKRQFRYGLIWGKSVKFGGQRVGLDHIVQDEDVLTIIKTRGA.................  369
DRG M. marburgensis       II...KEGSTVGDVCQKLHRDFVRKFRHARVWGSSVKFDGQKVGIDHVLNDEDVLRIIIKK...................  364
DRG M. barkeri            IV...MSGTNIGQICDRLHRDFRRKFRYAQVWGASAKHPGQRVGIEHRMQDEDVLTIIIQK...................  364
DRG M. vulcani            IV...RNGVTVGDICDHLHRDFRRKFRYAQVWGESAKHPGQRAGLDHVLADKDLLTLIIAK...................  363
DRG M. conradii           IM...RKGCTVGDVCDRLHKDFRRKFRYARVWGNSAKHAGQRVGLDHQLEDGDILTIIIQK...................  361
DRG H. archaeon           IV...KRDSTIRDICDKIHRSFKSQFRYAVVSGKSAKHPNQRVGLDHVIQDGDVITIIVKKF..................  371
DRG C. archaeon           IT...RDGSTIGDICDKLHRNMRKDFRYAMVWGKSVKFGGQRVGITHVLQDEDVLTIIKSR...................  366
DRG A. sulfaticallidus    II...KRGMTVGDVCKKLHKDMYRNFRYARVIGKSAKFKEQRVGIDHVLEDGDILTIYA.....................  354
DRG1 X. laevis            VLP..CSHTAAEDFCTKIHKNLIKEFKYALVWGSSVKHNPQKVGKDHVLEDEDVIQIVKK....................  367
DRG1 S. pombe             ILR..TGHSTVEDFCNNIHSSIKSQFKHAYVWGKSVPYPGMRVGLSHVLLDEDVVTIVKK....................  366
DRG1 S. cerevisiae        VLR..SDRCSVKDFCNQIHKSLVDDFRNALVYGSSVKHQPQYVGLNHILEDEDVVTILKK....................  369
DRG1 G. gallus            VLP..YCKTTVEDFCMKIHKNLIKDFKYALVWGSSVKHNPQKVGKDHTLEDEDVIQIVKK....................  367
DRG1 R. norvegicus        VLP..YSRTTVEDFCMKIHKNLIKEFKYALVWGLSVKHNPQKVGKDHTLEDEDVIQIVKK....................  367
DRG1 O. sativa           IVS..SKKKTVEDFCNRIHKDMVKQFKYALVWGSSVKHKPQRVGKEHELEDEDVVQIIKKV...................  369
DRG1 H. sapiens          VLP..YSRTTVEDFCMKIHKNLIKEFKYALVWGLSVKHNPQKVGKDHTLEDEDVIQIVKK....................  367
DRG1 D. melanogaster     VLH..NERTSIEDFCNKLHRSIAKEFKYALVWGSSVKHQPQKVGIEHVLNDEDVVQIVKKV...................  368
DRG1 D. rerio            VLP..DGRTAVEDFCLKIHKNLIKEFKYALVWGSSVKHNPQKVGKDHVMEDEDVIQLVKK....................  366
DRG1 C. elegans          VLN..AERKSIEDLCTKIHKSLQKDFKCALVWGASAKHNPQRVGRDHVLIDEDVVQVIKKV...................  366
DRG1 A. thaliana         ILS..SKKRTVEDFCIRIHKDMLKQFKYALVWGSSAKHKPQRVGKEHELEDEDVVQIVKKI...................  369
DRG2a A. thaliana        VLSADRGGCTVEDFCNQVHRTLVKDMKYALVWGTSARHYPQHCGLFHHLEDEDVVQIVKKKVREEGGRGRFKSHSNAPAR  387
DRG2b A. thaliana        VLSSDRGGCTVEDFCNHVHRTLVKDMKYALVWGTSTRHNPQNCGLSQHLEDEDVVQIVKKKERDEGGRGRFKSHSNAPAR  387
DRG2 X. laevis            IM...RKGASVEHVCHRIHRTLTSQFKYALVWGTSTKYSPQRVGLTHNMEHEDVIQVFKK....................  364
DRG2 S. pombe             IV...RKGSTIEQVCNRIHRTLAEQLKYALVWGTSAKHSPQVVGLNHRVEEGDVVTIVTK....................  363
DRG2 S. cerevisiae        VV...RNNSTIGDLCHGIHRDFKDKFKYALVWGSSAKHSPQKCGLNHRIDDEDVVSLFAK....................  368
DRG2 R. norvegicus        IL...RKGASVEHVCHRIHRSLASQFKYALVWGTSTKYSPQRVGLTHTMEHEDVIQIVKK....................  364
DRG2 O. sativa           VLSTDRGGCTVEDFCNHIHRSLVKDVKYVLVWGTSARHYPQHCGLGHVLQDEDVVQIVKKKEKEDGGRGRFKSHTNAPAR  387
DRG2 H. sapiens          IL...RKGASVEHVCHRIHRSLASQFKYALVWGTSTKYSPQRVGLTHTMEHEDVIQIVKK....................  364
DRG2 D. rerio            IM...RRAASVKHVCHRIHRTLASQFKYALVWGTSTKYSPQRVGLTHIMEHEDVIQIVKK....................  364
DRG2 G. gallus           IL...RKGASVEHVCHRIHRSLASQFKYALVWGTSTKYSPQRVGLTHMMEHEDVIQIVKK....................  364
DRG2 D. melanogaster     IL...RKGVSVEHVCHAIHRTLAAQFKYALVWGTSTKYSPQRVGIAHVMADEDVIQVVKK....................  363
DRG2 C. elegans          IL...RGGATIEHCCHALHRSIAAQLRYAIVWGTSTKFSPQRVGLHHKLDHEDVIQIVKK....................  366
```

DRG N. archaeon	....	369
DRG M. marburgensis	....	364
DRG M. barkeri	....	364
DRG M. vulcani	....	363
DRG M. conradii	....	361
DRG H. archaeon	....	371
DRG C. archaeon	....	366
DRG A. sulfaticallidus	....	354
DRG1 X. laevis	....	367
DRG1 S. pombe	....	366
DRG1 S. cerevisiae	....	369
DRG1 G. gallus	....	367
DRG1 R. norvegicus	....	367
DRG1 O. sativa	....	369
DRG1 H. sapiens	....	367
DRG1 D. melanogaster	....	368
DRG1 D. rerio	....	366
DRG1 C. elegans	....	366
DRG1 A. thaliana	....	369
DRG2a A. thaliana	IADREKKAPLKQ	399
DRG2b A. thaliana	IADREKKAPLKQ	399
DRG2 X. laevis	....	364
DRG2 S. pombe	....	363
DRG2 S. cerevisiae	....	368
DRG2 R. norvegicus	....	364
DRG2 O. sativa	ISDREKKAPLKT	399
DRG2 H. sapiens	....	364
DRG2 D. rerio	....	364
DRG2 G. gallus	....	364
DRG2 D. melanogaster	....	363
DRG2 C. elegans	....	366

X non-conserved
X similar
X $\geq$ 50% conserved
X all match
